# Endoplasmic Reticulum Stress of Gut Enterocyte and Intestinal Diseases

**DOI:** 10.3389/fmolb.2022.817392

**Published:** 2022-03-24

**Authors:** Han Gao, Chengwei He, Rongxuan Hua, Yuexin Guo, Boya Wang, Chen Liang, Lei Gao, Hongwei Shang, Jing-Dong Xu

**Affiliations:** ^1^ Department of Physiology and Pathophysiology, School of Basic Medical Sciences, Capital Medical University, Beijing, China; ^2^ Department of Clinical Medicine, School of Basic Medical Sciences, Capital Medical University, Beijing, China; ^3^ Department of Oral Medicine, School of Basic Medical Sciences, Capital Medical University, Beijing, China; ^4^ Undergraduate Student of 2018 Eight Program of Clinical Medicine, Peking University Health Science Center, Beijing, China; ^5^ Department of Biomedical Informatics, School of Biomedical Engineering, Capital Medical University, Beijing, China; ^6^ Experimental Center for Morphological Research Platform, Capital Medical University, Beijing, China

**Keywords:** endoplasmic reticulum stress, unfolded protein response, cell death, colon cancer, treatment target, inflammatory bowel disease

## Abstract

The endoplasmic reticulum, a vast reticular membranous network from the nuclear envelope to the plasma membrane responsible for the synthesis, maturation, and trafficking of a wide range of proteins, is considerably sensitive to changes in its luminal homeostasis. The loss of ER luminal homeostasis leads to abnormalities referred to as endoplasmic reticulum (ER) stress. Thus, the cell activates an adaptive response known as the unfolded protein response (UPR), a mechanism to stabilize ER homeostasis under severe environmental conditions. ER stress has recently been postulated as a disease research breakthrough due to its significant role in multiple vital cellular functions. This has caused numerous reports that ER stress-induced cell dysfunction has been implicated as an essential contributor to the occurrence and development of many diseases, resulting in them targeting the relief of ER stress. This review aims to outline the multiple molecular mechanisms of ER stress that can elucidate ER as an expansive, membrane-enclosed organelle playing a crucial role in numerous cellular functions with evident changes of several cells encountering ER stress. Alongside, we mainly focused on the therapeutic potential of ER stress inhibition in gastrointestinal diseases such as inflammatory bowel disease (IBD) and colorectal cancer. To conclude, we reviewed advanced research and highlighted future treatment strategies of ER stress-associated conditions.

## 1 Introduction

Due to the “lace-like” characteristics of the reticulum in the ground substance of cells grown in tissue culture by electron microscope (Qi and Chen, 2019) along with its closeness to the medial side of the cytoplasm, in 1945, the name endoplasmic reticulum was given (Porter and Thompson, 1948; Porter and Kallman, 1952). A cystic, vesicular, and tubular endoplasmic reticulum (ER) is part of the ER structure-derived membranous compartments and forms a continuous omentum system containing almost all eukaryotic cells. Along with that, the endoplasmic reticulum (ER) is the primary organelle responsible for regulating transmembrane and soluble secretory proteins processing, folding, assembly, modification, and lipid biosynthesis. This is also an organelle responsible for most transmembrane for its crowded membranous structures. Koch et al. (1986) discovered evidence for the most abundant calcium-binding glycoprotein located in ER for the first time of progression, suggesting that the major role of ER *in vivo* involves intracellular Ca^2+^ homeostasis.

Surprisingly, many studies have shown that cell stress response is linked to endoplasmic reticulum (ER) stress. The following experimental results supported this inference. First, ER dilated when faced with stresses such as heat (Chrispeels and Greenwood, 1987), dehydration (de la Pena de Torres, 1978), water overloading (de la Pena de Torres, 1978), fasting, cortisol injections, reserpine injections, restraint, spinal cord transaction, immersion in hot water, exposure to cold, and forced muscular exercise in a revolving drum (Salas et al., 1980). In addition, it has been discovered that many genes encode proteins located in ER, including the 78 kDa glucose-regulated protein GRP78 (also called BiP for binding immunoglobulin protein), the 94-kDa glucose-regulated protein (GRP94/ERp99), and ERp72, and some of the members of the stress protein family listed above have been increased in stress condition by a range of bioinformatic methods (Collins and Hightower, 1982; Welch et al., 1983; Lee et al., 1984). As countless investigations corroborated, the Hsp70 chaperone GRP78 works in concert to ensure the correct folding of proteins targeted for extracellular secretion (Haas and Wabl, 1983; Pelham, 1986), as well as binding proteins without being correctly folded and assembled to lead them to degradation pathways within the ER (Ng et al., 1990; Gething and Sambrook, 1992). When cells respond to turbulence from both internal and external environments, the function of ER that transfers transmembrane proteins and maturation of most secreted posttranslational modifications proteins molecules to cytoplasm will not work. This results in the ER becoming paralyzed due to the accumulation of folded/unfolded proteins. Then, the unfolded protein response (UPR) activates, resulting in a complex cellular response including the upregulation of abnormal protein degradation in the ER, with the goal of resolving the ER stress subsequently.

Served as three main sensors of UPR with the responsibility for transmitting messages to the rest of cells (Ron and Walter, 2007), the serine/threonine-protein kinase/endoribonuclease inositol-requiring enzyme 1 (IRE), protein kinase RNA-like ER kinase (PERK), and activating transcription factor 6 (ATF6) are crucial ER-resident transmembrane proteins. It is universally acknowledged that UPR is an integrated ER stress response pathway coordinated by three distinct pathways: the IRE-XBP1, the PERK-eIF2ɑ-ATF4, and the ATF6 pathway. The IRE/XBP1 pathway is the most conserved branch of the UPR, which facilitates the synthesis of ER chaperones to directly support ER protein folding (Lee et al., 2003b). The second one is the PERK-eIF2ɑ-ATF4 pathway, which suppresses translation to reduce the burden of ER (Harding et al., 1999). Lastly, ATF6 is a cryptic transcription factor entering the nucleus to upregulate target genes encoding chaperones transcriptionally.

To summarize, cells respond to misfolded proteins via three arms of the UPR. However, under conditions of sustained activation of ER stress state, the UPR switches modes from pro-survival to pro-apoptosis (Lu et al., 2014). As ER stressors themselves can cause cell death, such as apoptosis, pyroptosis, autophagy-dependent death, autophagy, and necrosis, they have attracted the attention of notable scientists. Therefore, this study aims to review current discoveries on ER stress and further analyze the correlation between ER stress and intestinal diseases to find a novel breakthrough point and therapeutic targets in ER stress-correlative-associated gastrointestinal disorders.

## 2 ER Stress and Its Overview

It is established that various exogenous and endogenous stresses such as hypoxia, starvation, infections, calcium depletion, acidosis, hyperglycemia, intoxication (Todd et al., 2008; Hetz, 2012), oxidized lipids, pathogen infection (Ye et al., 2011), salt stress (Liu et al., 2007), and heat stress (Liu and Howell, 2010), can evoke ER stress. Recent studies indicate that endogenous changes from cellular differentiation to profound metabolic induce the perturbations in ER function, which results from the accumulation of unfolded and misfolded proteins in the ER and triggers the unfolded protein response, also known as ER stress (Bettigole and Glimcher, 2015). From this, ER stress will initiate endoplasmic reticulum-related reactions by inducing a coordinated response, for instance, unfolded protein response (UPR), endoplasmic reticulum overload response (EOR), and sterol regulation reaction. The response mentioned above belongs to a protective counter-measure that reestablish homeostatic balance and promote survival by eliminating chronically misfolded protein or incorrectly folded proteins (Sitia and Braakman, 2003; Walter and Ron, 2011).

We initially summarized that ER stress responses are complex, involve multiple tissues and organs, and include corresponding functional changes. Thus, we addressed related UPR issues in this study. With the accumulation of unfolded and misfolded protein, UPR will be performed to restore homeostasis (Iurlaro and Munoz-Pinedo, 2016) by alleviating the burden of accumulated global protein synthesis and upregulating the ER-associated protein degradation (Ma and Hendershot, 2002; Todd et al., 2008).

## 3 ER Stress and Its Molecular Mechanism of Signaling Pathway

Herein, we summarize the active role played by ER stress in three signaling pathways and its mechanism of action in regulating these signaling molecules. The concept of ER stress being linked to dysfunctions of tissues and cells is generally accepted. So far, there are three crucial sensors: PERK (Ohoka et al., 2005), IRE1(Urano et al., 2000; Kouroku et al., 2007), and ATF6. They are activated and have been recognized in the UPR process. Under the physiological state, ER stress sensors are maintained in an inactive condition within the ER membrane through association with the chaperone protein, glucose-regulated protein 94 (Grp97), and GRP78 (Hamman et al., 1998; Malhotra and Kaufman, 2007; Tabas and Ron, 2011). The chaperone, GRP78, is one of the most abundant proteins within the ER that can enter the ER lumen, bind to a large variety of nascent polypeptides, and facilitate their translational folding correctly (Mori, 2000). Because unfolded substrates are more prone to bind GRP78 than the sensors, once ER stress is triggered, GRP78 will be sequestrated by the unfolded substrates to initiate UPR signaling. UPR signaling is a major pathway whereby cells respond to ER stress to reduce the synthesis of the general protein and restore new protein homeostasis (Clarke et al., 2014).

### 3.1 Biological Characteristic of IRE1

Cells harbor surveillance mechanisms to monitor protein-folding status and elicit adaptive responses to adjust protein-folding capacity. In other words, this mechanism of targeting protein quality control substrates expands the code that cells utilize to recognize aberrant proteins as it senses the state of translation rather than the folding state of the nascent chain. IRE1 belongs to type I transmembrane proteins kinase inositol-requiring enzyme 1, is the most evolutionary conserved among the sensors, remarkably exhibits similar mechanistic aspects shared between yeast and mammals, and has two paralogues encoded by nucleus signaling 1 and 2 (ERN1 and ERN2), respectively (Tirasophon et al., 1998; Wang et al., 1998; Iwawaki et al., 2001), IRE1α and IRE1β, in mammals. Structurally, the IRE1 can be divided into the three sub-compartments: an N-terminal luminal domain, a single-pass transmembrane spanning segment, and a cytosolic region subdivided into a Ser/Thr protein kinase domain (Tirasophon et al., 1998; Walter and Ron, 2011) and C-terminal endoribonuclease (RNase) domain (Hollien and Weissman, 2006). IRE1α was found and served as a positive control for detecting a functionally related ER-resident protein. Nonetheless, IRE1β was detectable by immunoblot of lysates prepared from purified microsomes from the small intestine, establishing specifically expressed in digestive tissues. And its switching components play a great part in pro-survival and pro-cell death signaling complexes. IRE1α regulates the dynamic signaling of the UPR. After accumulating unfolded or misfolded proteins in cells, IRE1α dissociates from GRP78, triggering IRE1α’s dimerization and trans-autophosphorylation mechanisms (Iurlaro and Munoz-Pinedo, 2016). Current evidence indicates that the oligomerization is crucial for IRE1 activation as it allows for trans-autophosphorylation and allosteric activation of its RNase domain by binding of ATP or ADP in the active site of the kinase. Upon activation, IRE1α cleaves the 26 pairs of base-pair fragments from mRNA of encoding the transcription factor X-box binding protein 1 (XBP1), resulting in the unspliced XBP1 (XBP1u) forming spliced XBP1 (XBP1s) that encodes a wide variety of UPR target genes (Sidrauski and Walter, 1997; Yoshida et al., 2001; Meyerovich et al., 2016). XBP1u is a short-lived protein that is rapidly degraded by proteasomes (Lee et al., 2003a). On account of a basic leucine region zipper (bZIP) transcription factor, XBP1s are able to control protein folding, secretion, and phospholipid synthesis. With the regulation of ER chaperone protein-coding, XBP1s is a member of protein degradation in ER cavity (Park et al., 2017) and is commonly used as a readout of UPR activation (Campbell et al., 2013).

**FIGURE 1:**
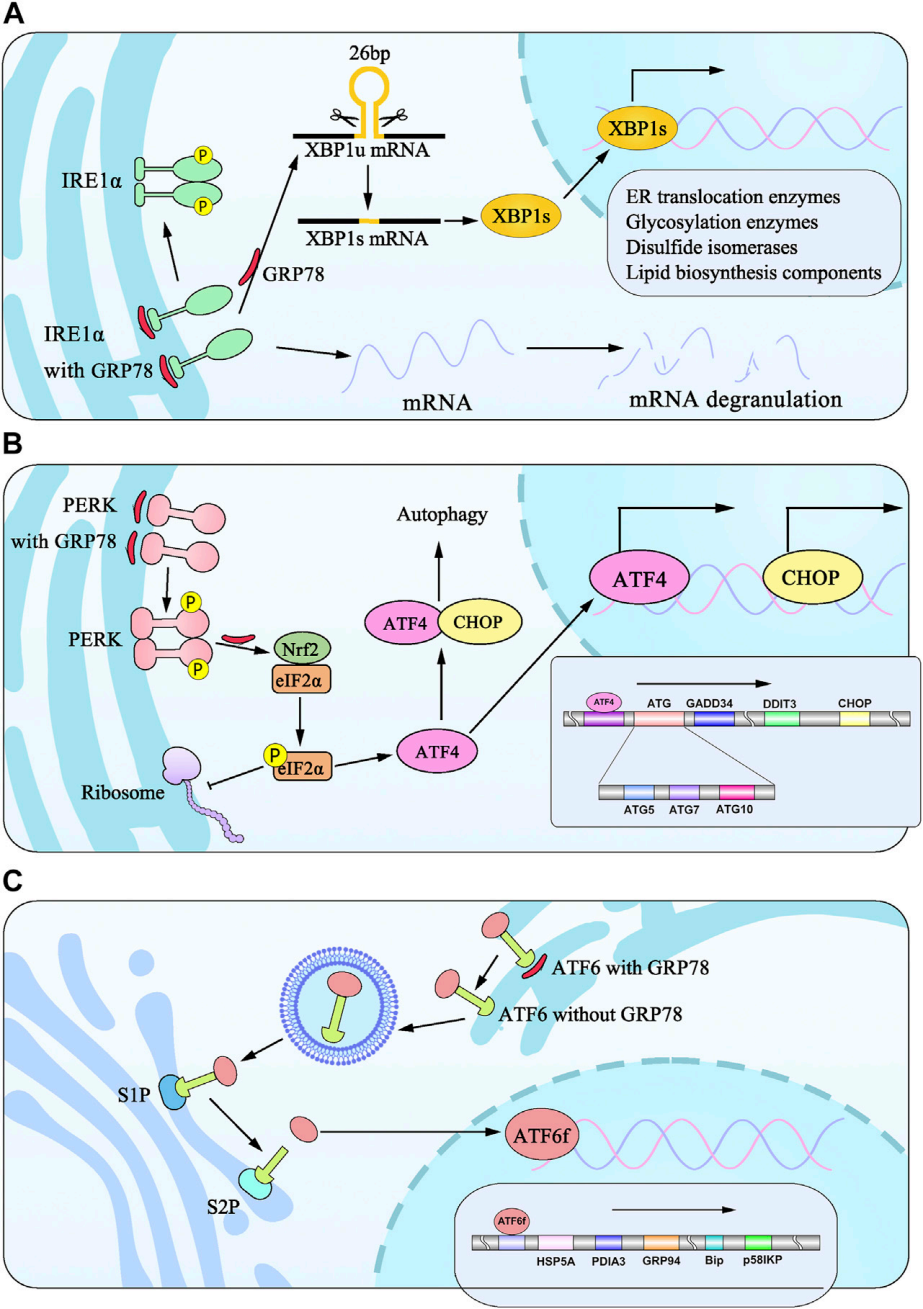
Model diagram of signaling pathways from three ER transmembrane stress sensors during UPR induced by ER stress. **(A)** IRE1α isolates from BiP undergo dimerization and phosphorylation to splice 26bp from XBP1 into XBP1s, which translocates to the nucleus and induces the transcription of target genes. **(B)** PERK signaling is initiated through PERK dimerization and autophosphorylation of the cytosolic PERK kinase domain. eIF2α phosphorylation is elicited to attenuate global protein synthesis. eIF2α promotes translation of ATF4, which translocates to the nucleus to regulate the expression of target genes and cooperates with CHOP to participate in ER stress-induced apoptosis pathways. **(C)** ATF6 dislocates from BiP translocates to Golgi apparatus, which is cleaved by proteases at S1P and S2P sites into ATF6f and promotes the transcription of genes.

Noting this, activation of the IRE1/XBP1 pathway, the most conserved branch of the UPR, can upregulate the expression of ER translocation enzymes, glycosylation enzymes, disulfide isomerases, and boost transcriptionally genes involved in lipid biosynthesis components, whereby protein synthesis and accumulation in the ER cavity can be effectively reduced. Specifically, under the condition of severe ER stress, the IRE1 endonuclease activity can also degrade mRNAs (Hollien and Weissman, 2006; Hollien et al., 2009), thereby providing a way to reduce ER overload and playing a paramount role in regulated IRE1-dependent mRNA decay (RIDD). Hollien and Weissman (2006) reported that it is likely to produce additional insights into the biological outcomes of ER stress beyond the simple degradation of unwanted mRNAs. Moreover, overexpression of IRE can lead to cleavage of 28S rRNA (Iwawaki et al., 2001). Contrary to previous evidence, Jeffery S and his colleagues unambiguously confirmed that the IRE1 function is required to protect cells from ER stress. In addition, they also established that IRE1^−/−^ are prone to death from administrated tunicamycin (TM) or β-mercaptoethanol, autophagy-inducing agents, which can lead to accumulation of unfolded proteins in the JC103 and CS165 cultures growing in the middle phase (Cox et al., 1993). It is worthy of noting that Jonathan H. Lin et al. demonstrated that human embryonic kidney (HEK) 293 cells pretreated with TM or thapsigargin (TG) (the SERCA ATPase sarco-endoplasmic reticulum Ca^2+^-ATPases inhibitor) exhibited ER stress with an evaluation of Xbp-1s mRNA level detected by reverse transcription-polymerase chain reaction (RT-PCR) and its protein levels tested by western bolting, which indicated IRE1 activation. To further assess the effect of IRE, through selectively upregulating IRE1 activity by treating HEK293 cells with adenosine triphosphate (ATP) analog 4-amino-1-tert-butyl-3-(1′-naphthylmethyl) pyrazolo (Kim et al., 2020) pyrimidine (1NM-PP1), results indicated that the number of surviving cells is much higher (Lin et al., 2007). Furthermore, normal human colon epithelial cell line (NCM-460) cell viability was significantly reduced upon IRE-1 RNA interference. At the same time, overexpression of IRE-1 decreased cell apoptosis rate and rescued IL-6-induced cell apoptosis (Zhang B. et al., 2021). The above data all powerfully evidenced that IRE1 is responsible for promoting cell viability (Figure 1A).

### 3.2 PERK and Its Biological Properties

PERK is encoded by the *perk* gene that phosphorylates the α-subunit of eukaryotic translation-initiation factor 2 (EIF2S1/eIF-2α) in chromosome 2 p11.2 88556741–88627576. Also known as EIF2AK3, PERK belongs to serine/threonine-protein kinase, contains a distinctive amino-terminal region 550 residues in length, and possesses a similar structure to IRE1. It belongs to type I transmembrane protein (Shi et al., 1998; Harding et al., 1999; Bertolotti et al., 2000). Intriguingly, PERK binds to GRP78 via its N-terminal domain and dissociates depending upon forming its homodimers in case of ER stress. The C-terminal region is cytosolic and contains the kinase domain and autophosphorylation sites (Harding et al., 1999), activating the downstream signaling pathway (Kikuchi et al., 2016). PERK is a kinase occurring at Tyr615. Upon activation, PERK autophosphorylates (Su et al., 2008) and becomes able to efficiently phosphorylate eIF2α to attenuate transient global translation (Shi et al., 1998; Harding et al., 1999; Harding et al., 2000b). At length, the activated PERK triggers the transcription factor nuclear factor-erythroid 2-related factor 2 (Nrf2), thereby inducing antioxidant proteins. It also binds to the eIF2α to promote eIF2α phosphorylation at the serine 51 residue, as Shi et al. reported in cell lines and Sprague–Dawley rats models. Aside from that, eIF2α also plays a pivotal role in the early steps of mRNA translation from nematodes to mammals through identifying PEK homologs belonging to *Caenorhabditis elegans* and pufferfish *Fugu rubripes*. In fact, eIF2α phosphorylation is essential for cell survival. Through the mutation of eIF2αphosphorylation at Ser51 on the α subunit, mutant eIF2α cells showed a larger protein synthesis and necrosis by flow cytometry than wild-type cells (Scheuner et al., 2001). It is attested that phosphorylation of eIF2α suppresses 80S ribosome assembly, attenuating protein synthesis and misfolded protein overload in ER (Pain, 1996; Cao et al., 2013). In conclusion, that summarizes the recent progress of research focusing on cell survival by reducing protein synthesis following ER stress. A detailed understanding of this mechanism can be seen in Figure 1B.

It is well known that multiple signaling pathways are involved in protein synthesis, and activation of the PERK pathway suppresses the production of the proteins under ER stress to restore cellular homeostasis. Numerous experimental studies have demonstrated that phosphorylation of eIF2α regulates translation of the ATF4 mRNA by activating multiple upstream open reading frames (Harding et al., 2000a; Lu et al., 2004; Vattem and Wek, 2004). ATF4, known as an efficiency transcription factor, induces the expression of antioxidant- and amino acid-related genes. It has been found to play a role in protein synthesis and secretion, such as DNA damage-inducible transcript 3 (DDIT3) DNA damage-inducible 34 (GADD34) (Harding et al., 2003; Han et al., 2013). DDIT3, as a trans-activator of transcription genes, can actively regulate apoptosis. It should also be noted that ATF4 and dephosphorylates eIF2α contribute to upregulating GADD34 identified as a participant for translational recovery (Novoa et al., 2001; Ron, 2002) (Box1). Alongside, the influence on phosphorylation of eIF2α and inhibition of mRNA translation is transient (Pavitt and Ron, 2012). Moreover, ATF4 induces a second transcription factor, C/EBP homologous protein (CHOP, also known as GADD153), which promotes autophagy as described in Box2.

#### 3.2.1 Box 1 ATF4

ATF4 (activating transcription factor 4) belongs to the DNA-binding protein family of transcription factors, locates on chromosome 22 q13.1 39519695–39522685, and is highly regulated by eIF-2α/EIF2S1 phosphorylation. ATF4 is also an activator of the stress-responsive gene transcription factors, suggesting that the recovery of cellular function may be mediated by ATF4.

With the impact of ER stress, the expression of ATF4 is dependent on PERK activity and eIF2α phosphorylation, ATF4 level among *perk* ^−/−^ cells, and EIF2αS51A/S51A knock-in cells (which cannot be phosphorylated by eIF2α kinases). Along with that, cells overexpressing an eIF2α phosphatase have decreased significantly (Harding et al., 2000a; Novoa et al., 2001; Scheuner et al., 2001). Additionally, *Atf4*ΔIEC mice have developed spontaneous enterocolitis and colitis of greater severity than control mice after administration of DSS (Hu et al., 2019).

Apart from acting as a master transcriptional regulator of the ER stress, ATF4 regulates various genes, including those involved in amino acid transporters and cellular redox control genes (Harding et al., 2003).

It should also be noted that ATF4, a key transducer (Rutkowski and Hegde, 2010; Hetz, 2012), not only directly induces expression of GADD34, which further contributes to translational recovery and dephosphorylating eIF2α (Novoa et al., 2001; Ron, 2002), but also drives the transcription of genes involved in autophagy such as ATG genes, Atg7, Atg10, and Atg5 (B'Chir et al., 2013).

#### 3.2.2 Box 2 CHOP and Its Functions

CHOP (C/EBP-homologous protein), also known as growth arrest and DNA damage-inducible gene 153 (GADD153), is highly expressed in the case of consecutive ER stress containing basic leucine zipper (bZIP) domains. bZIP is a transcription factor by which CHOP forms heterodimers with C/EBP family members (Ron and Habener, 1992; Wang et al., 1996).

CHOP regulates more than 200 genes encoding proteins promoting autophagy (Marciniak et al., 2004; Rouschop et al., 2010). Experimental evidence strongly tied to apoptosis induced by ER stress shows that CHOP ^−/−^ displays attenuated death induced by TM, TG, and A23187, of which the conclusion obtained from comparing cells periodically by phase-contrast microscopy of mouse embryonic fibroblasts (MEF) derived from chop^−/−^ and chop^+/+^ genotypes (Zinszner et al., 1998). Recently, detailed investigations indicated that cell death apoptotic pathways induced by cell death-targeted treatment were not activated following the knockdown of CHOP (Dilly et al., 2020).

Mechanisms of CHOP-inducing apoptosis have been elucidated by focused studies on apoptosis-related genes, including apoptotic B cell lymphoma 2 (Bcl-2), GADD34, ER oxidoreductin 1 (ERO1ɑ), and tribbles-related protein 3 (TRB3) (Szegezdi et al., 2006). On the one hand, the levels of GADD34, a regulatory subunit of an eIF2α-specific phosphatase complex, can be mediated by CHOP via upregulating the transcription of ERO1α. This activates IP3R and promotes calcium release to mitochondria, thereby inducing cell apoptosis (Li et al., 2009; Xia et al., 2019). On the other hand, CHOP facilitates the expression of Bim that initiates the mitochondrial pathway of apoptosis, which realizes negative feedback on Bcl-2, playing a role in inhibiting mitochondrial outer membrane pore formation (McCullough et al., 2001). Intriguingly, ATF3, as one of the substrates of CHOP, can be coordinated to bind the promoter of the genes of TNF-related apoptosis-inducing ligand (TRAIL) receptor 1/2 (TRAIL-R2/DR4/5), facilitating their expression to initiate activation executioner caspase-8 leading to cell death.

### 3.3 ATF6

Activating transcription factor 6 (ATF6), a type II ER transmembrane protein with a molecular weight of 90 kDa, has two isoforms of proteins identified in mammalian cells: ATF6α and ATF6β (Han et al., 2013). Once ER stress occurs, ATF6 dissociates from GRP78, transfers to the Golgi apparatus, and is sheared into ATF6 fragments (ATFf) with an active N-terminal 50 kDa domain by site-1 protease (S1P) and site-2 protease (S2P), which possess the capacity for shearing in Golgi (Chen et al., 2002; Hong et al., 2004; Nadanaka et al., 2004; Shen et al., 2002). With the cytosolic basic leucine zipper (bZIP) transcription factor assistance, ATF6f can enter the nucleus through the nuclear membrane and regulate the transcription of the related genes involved in protein folding, such as HSP5A, PDIA3, GRP94, GRP78, and p58IPK (Kokame et al., 2001; Wang et al., 2000; Yoshida et al., 1998). The detailed process is illustrated in Figure 1C. Apart from that, via binding to cAMP-responsive elements (CRE) and ER stress-response elements (ERSE-1), ATF6f is involved in the ER-associated degradation (ERAD) pathway contributing to protein degradation (Yoshida et al., 2001; Chiang et al., 2012; Huang et al., 2018). Furthermore, ATF6 and PERK enhance the levels of CHOP expression, thereby leading to the induction of autophagy genes. Moreover, ATF6 is functionally crucial for the nucleation of autophagosomes, indicating that ATF6-mediated signaling plays a critical role in cell survival in response to ER stress (Szegezdi et al., 2006).

## 4 Cross Talk of Cell Death and ER Stress

Changes in cells’ fate after the impact of ER stress are diverse, depending on whether being mild, transient, or severe. Chances are alleviated to restore homeostasis if ER stress is mild or transient. In contrast, in the case of ER stress persisting overwhelmingly, cell manipulations may cause changes in gene expression or even cell death along with three parallel forms of cell death: apoptosis (Choi et al., 2019), pyroptosis (Ke et al., 2020), and autophagy (Kaser and Blumberg, 2010).

### 4.1 Apoptosis Induced by ER Stress

It is widely accepted that the fundamental pathways of UPR can trigger apoptosis in case of overwhelming ER stress. IRE-1α induces apoptosis by cleaving microRNAs regulating caspase-2 expression through the RNase activity (Hassler et al., 2012). Interestingly, *perk* ^−/−^cells exhibit disturbed ER morphology and Ca^2+^ signaling and are feeble in the ER-mitochondria contact sites. Details shown in Box3 indicate that PERK is uniquely enriched at the mitochondria-associated ER membranes (MAMs) (Fan and Simmen, 2019). It has been reported that antioxidant coordinator Nrf2 is a target of the PERK kinase activity (Harding et al., 2003). However, given the relatively few studies involving this mechanism, it will be fascinating to unravel this challenging question. In addition, PERK is involved in mediating X-linked apoptosis inhibitor of protein (XIAP), a member of the inhibitor of apoptosis (IAP) family of proteins repressing apoptotic cell death by activating degradation of caspase-3, caspase-7, and caspase-9 through ubiquitin-mediated protein degradation (Muaddi et al., 2010). XIAP expression is mainly upregulated by an eIF2α phosphorylated mechanism at the translational level, contributing to cell survival. While ATF4 may contribute to caspase activation by promoting XIAP degradation, in turn, it impedes this process. Most importantly, different pharmacological compounds, such as CHOP, eIF-2α, and c-Jun NH2-terminal kinase (JNK), are involved in UPR-induced apoptosis (details seen Box1). JNK (also known as stress-activated protein kinases, SAPKs) and caspases have also been involved in mediating apoptotic signals during ER stress (Ip and Davis, 1998). There currently is experimental evidence available to suggest the mechanisms. Upon ER stress, IRE1α determines the expression of JNK in wild-type fibroblasts and IRE1α^−/−^ fibroblasts (Urano et al., 2000). Furthermore, under conditions of severe ER stress, mRNA IRE1 decay may promote cell death by degrading the mRNAs of anti-apoptotic proteins, thus tipping the balance towards apoptosis (Logue et al., 2013).

#### 4.1.1 Box3 Mitochondria-Associated ER Membranes

**FIGURE 2:**
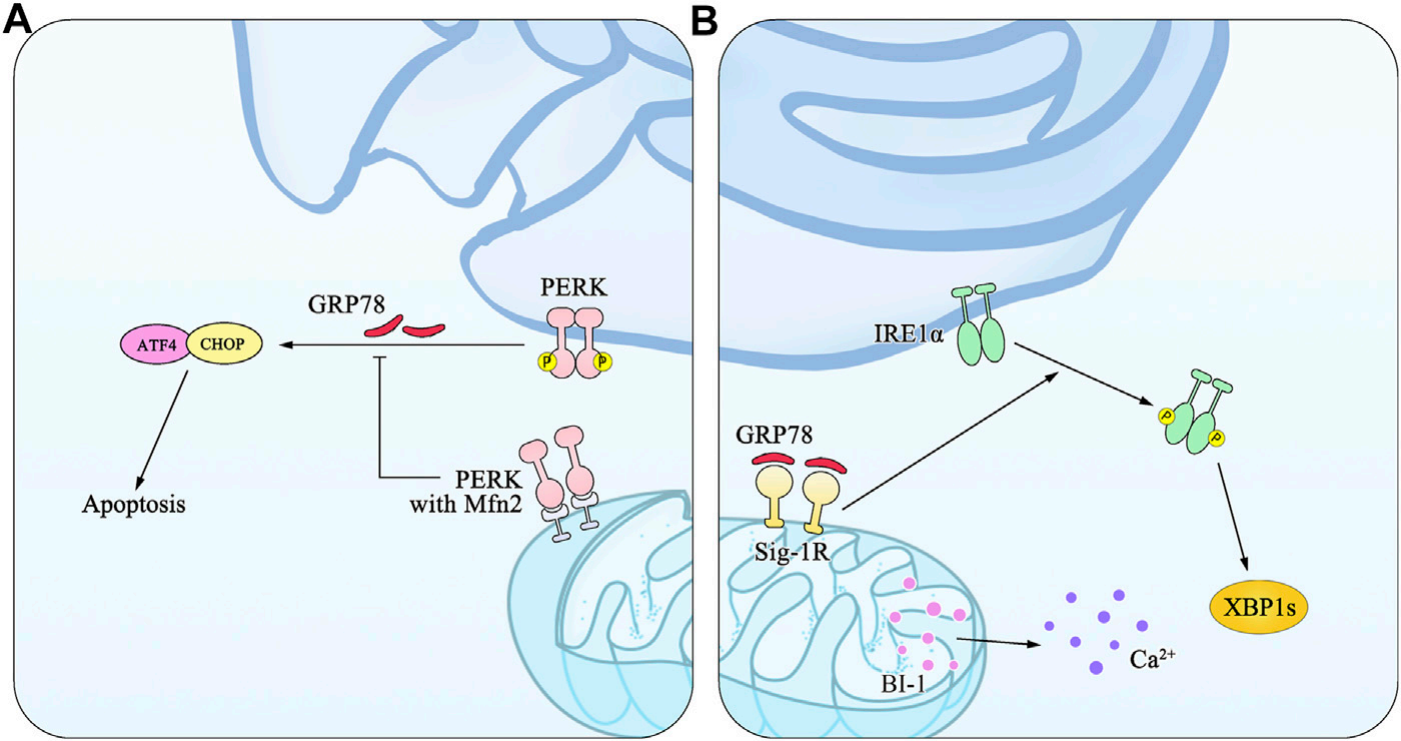
An illustration of MAMs and mitochondrial dysfunction in ER stress. MAMs are the ER membranes at the MERCs, which form a stable bridge between the ER membrane and the OMM, whose fusion is mediated by Mfn1, Mfn2, and Sig-1R. **(A)** Mfn2 is involved in alterations of mitochondrial morphology through binding to PERK and inhibiting PERK signaling. **(B)** As the local expression of chaperone proteins, Sig-1/BiP activates the IRE1/XBP1 signaling pathway via IRE1 dimerization. BI-1 resides on the MAMs and regulates mitochondrial Ca2+ concentration and apoptosis.

The sites of physical communication between the ER and mitochondria are defined as mitochondria-associated membranes (MAMs) (OMM) (Wieckowski et al., 2009; Vance, 2015). Dynamin-like GTPase mitofusin-2 (Mfn2), one of the multiple proteins assembled by MAMs, is involved in regulating mitochondrial fusion through binding to PERK to inhibit PERK signaling and mediating mitochondrial functions (Santel and Fuller, 2001; Munoz et al., 2013). Furthermore, PERK is enriched at MAMs where it facilitates the tethering of the ER to mitochondria and sensitizes cells to apoptosis, which is also proved in the Mfn2^−/−^ cells through several experimental models (Verfaillie et al., 2012; Munoz et al., 2013). The sigma 1 receptor (Sig-1R), local expression of chaperone proteins, is located at MAMs, and may bind to BiP to form a Sig-1R/BiP complex, through which it induces IRE dimerization and further activates the IRE1-XBP1 signaling pathway. MAMs are also known as multiprotein platforms, where Ca^2+^ releases are regulated by Bax-inhibitor-1 (BI-1). BI-1 is an antagonist of Bax that has a suppressive action on the IRE1-XBP1 signaling pathway. Consequently, the deficiency of BI-1 elicits IRE1 activation accompanied by its downstream enhancement (Lisbona et al., 2009). The detailed mechanism is illustrated in Figure 2.

### 4.2 Mitochondrial Pathway of Apoptosis-Induced ER Stress

**FIGURE 3:**
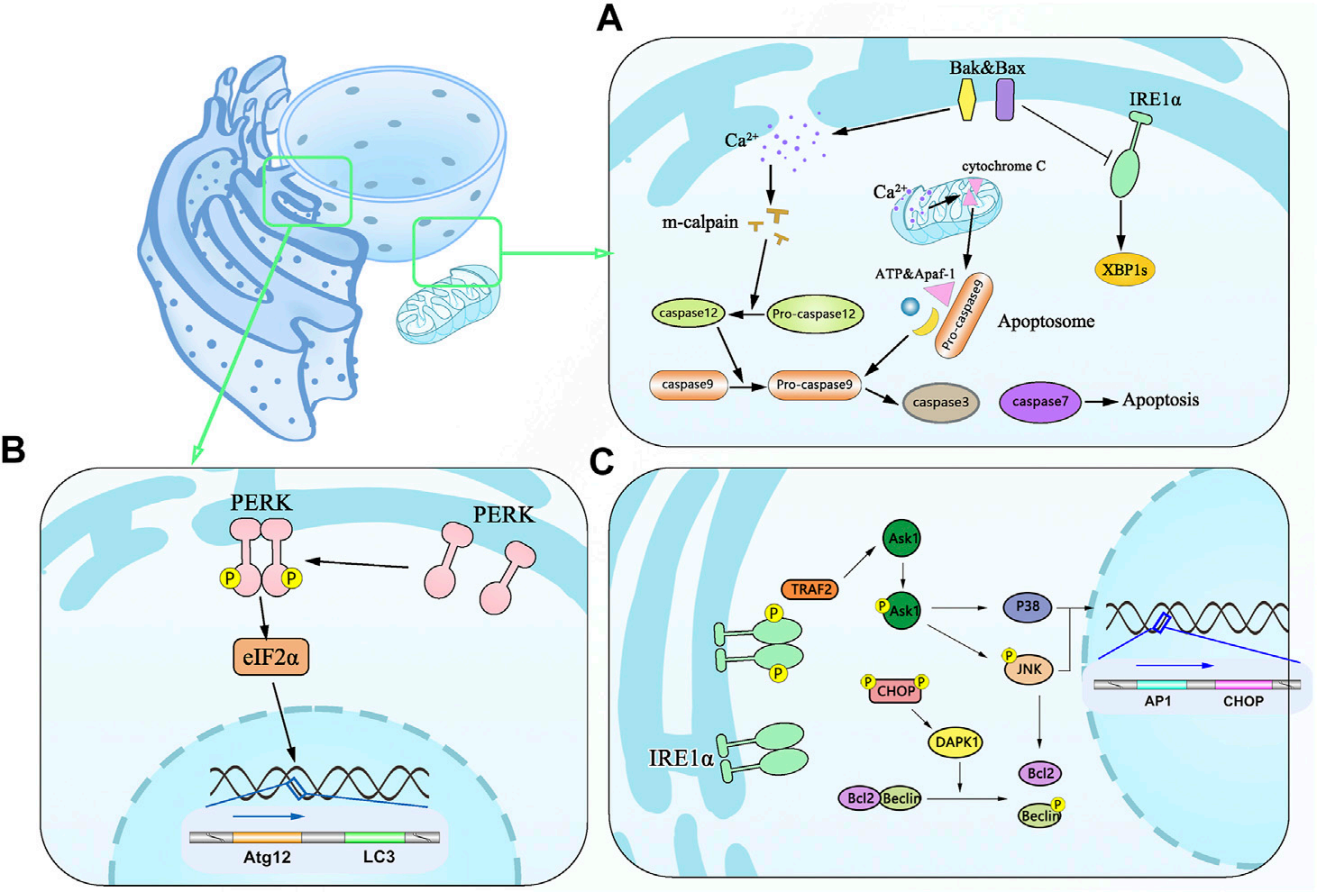
Schematic diagram of apoptosis associated with the mitochondrial pathway and autophagy induced by ER stress. **(A)** Effector proteins of Bax and Bak in apoptosis undergo conformational changes in active forms and oligomerize in the ER membrane leading to the efflux of Ca2+ from the ER to the cytoplasm and activating m-calpain to cleave and activate procaspase-12, which in turn activates caspase-3 and caspase-7. BAX and BAK are associated with the decreased expression of IRE1 and XBP1 and promote apoptosis. Ca^2+^ can be taken up by mitochondria leading to depolarization of the mitochondrial inner membrane and cytochrome c release, which binds Apaf-1, procaspase-9, and ATP into apoptosome and activates caspase-9 and caspase-3, then resulting in cell death. **(B)** Dimmerized PERK phosphorylates the translation initiation factor eIF2α and mediates the translation of Atg12 and LC3. **(C)** Activated IRE1 forms P-IRE1α/TRAF2 complex and initiates Ask1 as a stress-responsive in the JNK and p38 pathways. Phosphorylated JNK along with activated P38 enters the nucleus to promote translation of AP1 and CHOP.

Two principal signaling pathways initiate apoptosis. One is the exogenous or death receptor pathway, while the other is the mitochondrial pathway. Notably, the latter frequently occurs when the cells are captured under the UPR condition. B-cell lymphoma 2 (BCL-2) family proteins containing Bcl-2 associated X proteins (Bax) and Bcl-2 antagonists/killers (Bak), both localized to the mitochondrial outer membrane and the inner ER (Krajewski et al., 1993; Zong et al., 2003), are the major molecules of provoking the apoptosis via the mitochondrial pathway. Cells deficient for both BAX and BAK become resistant with the stimulation of TG, TM, and brefeldin A (Wei et al., 2001). Activated Bax/Bak can lead to apoptosis via two different pathways. Firstly, Bax/Bak change conformation from inactive forms to active forms and oligomerize in the ER membrane upon ER stress, localizing the ER to regulate ER Ca^2+^ levels in the reticular lumen (Scorrano et al., 2003; Zong et al., 2003). A high concentration of cytoplasmic Ca^2+^ activates *m*-calpain, leading to the cleavage and activation of procaspase-12 (Nakagawa and Yuan, 2000) along with the downstream caspase-12 and activation procaspase-9. This, in turn, provokes several downstream effector caspases (caspase-3 and caspase-7) (Morishima et al., 2002; Vannuvel et al., 2013). Many feedback mechanisms, as summarized in the above paragraph, are essential for accurate assessments of caspase and ER stress, yet Ca^2+^ in the mitochondrial pathway should not be ignored. Mitochondria take up Ca^2+^ from the cytosol, leading to depolarization of the mitochondrial inner membrane and the subsequent release of cytochrome c (Vannuvel et al., 2013), which can bind to Apaf-1, procaspase-9, and ATP to form apoptosome. This activates caspase-9, which causes the downstream executor to activate caspase-3, DNA fragmentation, to initiate cell death (Crompton, 1999; Shibue and Taniguchi, 2006). Apart from this, Bax and Bak have been found to interact directly with the cytosolic domain of IRE1 and decrease XBP1 expression (Hetz et al., 2006) (Lai et al., 2007). The main point can be summarized in Figure 3A: mitochondria are essential organelles responsible for cellular stress responsiveness.

### 4.3 Correlation Between Autophagy and ER Stress

There is mounting evidence that autophagy is an evolutionarily conserved process involving the regulation of cellular homeostasis via removing misfolded proteins and damaged organelles. Autophagy is the membrane trafficking pathway that delivers intracellular degraded material to lysosomes via the double-membrane vesicles, the autophagosomes (Inoue and Moriyasu, 2006; Liu and Bassham, 2012). Through the effect of ER stress, autophagy plays a protective role in a constitutive manner to enable the turnover of long-lived proteins, removal of damaged organelles, and misfolded proteins as a defense mechanism against pathogens (Yang et al., 2016). When the accumulation of damaged proteins in the ER has exceeded the repair capacity of ERAD, portions of the organelle can be specifically targeted for large-scale degradation through autophagy (Lajoie and Snapp, 2020). eIF2α phosphorylation induced by PERK works as a hotspot of stress-induced translation control (Kouroku et al., 2007; Liu et al., 2010; Talloczy et al., 2002). This effect is associated with overexpression of the autophagy-related gene Atg2 and LC3 *in vivo* and *in vitro*. Numerous *in vitro* studies have demonstrated that PERK/ATF4/CHOP signaling mediates the upregulation of LC3 and Atg5 to drive specific phagophore formation phenomena (Mizushima, 2005; Rouschop et al., 2010) (Figure 3B). Notably, a recent report has shown the effect of CHOP contributing to apoptosis through upregulating the expression of the death-associated protein kinase 1 (DAPK1). Furthermore, GRP78 overexpression is sufficient to induce UPR or autophagy through the IRE1 pathway after treatment with TM and dithiothreitol (Chan and Egan, 2005). Interestingly, some researchers believe that the cell autophagy signal transduction pathway induced by ER stress is IRE1 rather than PERK and ATF6. These challenges likely contribute to different conclusions in previous studies. We think these phenotype differences have been associated with different abundances in different tissues, partly due to differential ER stress models in different tissues.

The apoptosis mentioned above is regulated by the PERK/ATF4/CHOP protein family. However, the IRE1 modulatory role in autophagy and ER stress should not be ignored in the process. IRE1 binds TNF-receptor-associated factor-2 (TRAF-2) to form a complex that phosphorylates and activates the apoptosis signal-regulating kinase 1/MAP3K5 (ASK1), which in turn mediates the phosphorylation of ASK1 and induce c-Jun N-terminal kinase 1 (JNK/MAPK8/SAPK1) phosphorylation, thus leading to Bcl-2 activation. This ultimately causes autophagy (Urano et al., 2000; Talloczy et al., 2002; Ogata et al., 2006; Kouroku et al., 2007; Liu et al., 2010; Carreras-Sureda et al., 2017; Ishikawa et al., 2017). Prolonged activation of JNK-mediated stress is well known to instigate deleterious inflammation, leading to inflammation by triggering the secretion of proinflammatory chemokine (Kaser et al., 2008). Aside from TRAF2 binding to I-κ B kinase (IKK), activated NF-κB leads to the enhancement of pro-inflammatory TNF-α to facilitate stress-induced cell death (Hu et al., 2006; Li et al., 2011). To obtain support for the proposed mechanism and better understand the structural factors, including re-incarceration, a detailed illustration is shown in Figure 3C.

### 4.4 Correlation Between Pyroptosis and ER Stress

**FIGURE 4:**
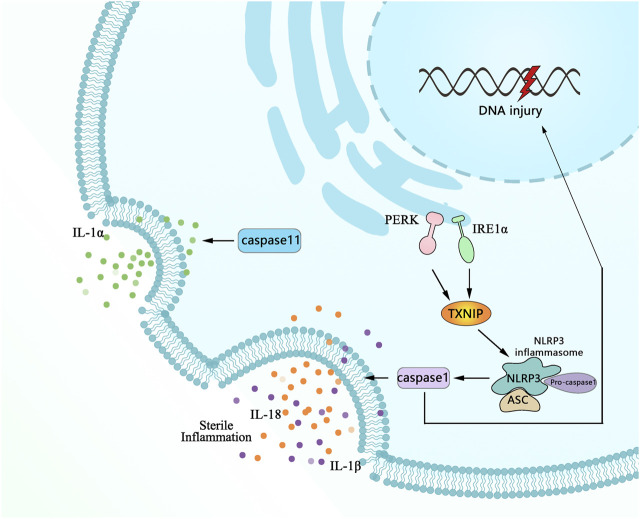
Schematic highlights of the complex network on multiple factors involved in signaling pathways for ER stress-induced pyroptosis. Following injury, pro-inflammatory mediators of proIL-1β, NLRP3, and caspase-11 are increased transcription. ProIL-1β and TXNIP facilitate the assembly and activation of the NLRP3 inflammasome that cleaves procaspase-1 to active caspase-1, which in turn leads to process maturation of the IL-1β and initiating pyroptosis with the feature of plasma membrane rupture and DNA fragmentation along with multifarious cytopathological changes.

Pyroptosis, a prominent form of programmed lytic cell death mediated by pro-inflammation, is the best-characterized response (Brennan and Cookson, 2000). For this reason, understanding the molecular mechanisms regulating sustained ER stress-induced pyroptosis has been the subject of intense research efforts to identify either cause or alter risk (Zhou et al., 2020). Recent studies have revealed that exposure to hypoxia directly triggers pyroptosis under ER stress, as shown by the high immunoreactive nucleotide-binding oligomerization domain-like receptor family members, containing pyrin domain 3 (NLRP3), inflammatory caspase-1, and IL-1β. These mediators were identified as critical mediators of pyroptosis, and the detailing process is described in Figure 4. The activation of the inflammasome can cause a necrotic form of cell death termed pyroptosis (Dong et al., 2015; Jorgensen et al., 2016; Man et al., 2017). Currently, the view of the structure-function relationship of NLRP3/caspase-1/IL-1β has been widely accepted, a primary inflammatory mediator in initiation pyroptosis is the NLRP3 inflammasome and caspase-1, the effector protease of the NLRP3 inflammasome, which is essential for pro-IL-1β and IL-18 secretion (Schroder and Tschopp, 2010; van de Veerdonk et al., 2011). There are several other approaches, such as thioredoxin, an oxidative stress mediator interacting protein (TXNIP) linked to NLRP3 inflammasome activation and serving as a marker for excessive ER stress and UPR activation (Cheng et al., 2019). More importantly, inhibiting ER stress with TXNIP siRNA and 4-phenyl butyric acid (PBA) prevents activation of NLRP3 inflammasomes (Luo et al., 2014) and so did IRE1αRNase specific inhibitor (STF-083010) (Ke et al., 2020).

## 5 Heat Shock Proteins and ER Stress

Heat shock proteins (HSPs), discovered in almost all organisms (Craig, 1985), are subdivided into distinct families according to their sequence homology and molecular weight: HSPH (former name HSP110), HSPC (HSP90), HSPA (HSP70), HSPD/E (HSP60/HSP10 and CCT (TRiC), DNAJ (HSP40) (the above so-called large HSPs), and HSPB (small HSP or sHSP, with the molecular weights of 12∼ 43 kDa) (Vos et al., 2008; Kampinga et al., 2009). Some of HSPs are expressed constitutively in normal cells. Likewise, the expressions of other HSPs are robustly enhanced in response during adverse conditions. Extensive studies have corroborated that HSPs, as organellar proteins (Krishna and Gloor, 2001; Emelyanov, 2002), guard the proteome against misfolding and aggregation of denatured proteins (Johnston et al., 1998).

The sHSPs share a homologous amino acid sequence called the “α-crystallin domain” (Caspers et al., 1995), which can form large oligomers due to environment-unfriendly conditions (Arrigo, 1990; Klemenz et al., 1991; Inaguma et al., 1993). Thereby, it is generally accepted that sHSPs are responsible for relieving ER stress alleviation via multiple regulatory pathways (Kim et al., 2008; Morimoto, 2008).

### 5.1 HSP27 (HSPB1)

In humans, HSP27 is ubiquitously expressed in all tissues, predominantly located in the cytosol and smaller portions in the membrane and cytoskeletal fractions. Full-length HSP27 comprises 205 residues but self-assemble into large, polydisperse oligomers depending strongly on external conditions (Jovcevski et al., 2015; Alderson et al., 2020). There is a general agreement on the correlations between HSP27 and ER stress. It was revealed that pretreatment of U373 MG human glioma cells with ER inducers, TM or TG, high expressions of HSP27, and αB-crystallin were induced, and the phosphorylation of HSP27 was upregulated by mediating activation of the p38 MAP kinase pathway (Ito et al., 2005). Notably, it is postulated that HSP27, as a cellular stress sensor, not only is a potent inhibitor of apoptosis responding to diverse stress (Rogalla et al., 1999; Samali et al., 2001) but also attenuates ER stress-induced intrinsic apoptosis through downregulated expression of annexin, procaspase-9/procaspase -3, and caspase-3/caspase-7, due to their vital role in ERK-mediated phosphorylation and the proteasomal degradation of BIM (Kennedy et al., 2017). Endoplasmic reticulum protein 29 (ERp29) involved in ER stress is closely related to HSP27. Some evidence confirmed that overexpression of ERp29 in cancer, including breast cancer (Mkrtchian et al., 2008), nasopharyngeal carcinoma (Wu et al., 2012), and pancreatic cancer (Mori-Iwamoto et al., 2007), can raise the expression of Hsp27, thereby decreasing apoptosis. It is worth mentioning that HSP27 involves regulating multiple processes in cells and suggesting that HSP27 represents a new therapeutic target for some special diseases (details seen Table 1). However, further investigations are required to reveal the more detailed associations between HSP27 and ER stress.

**TABLE 1:** Small and large HSPs involved in diseases.

1	S	Name	Disease	References
2	Small HSPs	HSP27	Colorectal cancer; breast cancer; oral tongue squamous cell carcinoma	Wang et al. (2009), Yu et al. (2010), Thuringer et al. (2015), Wang et al. (2020)
3		α-Crystallins	Tumors; neurodegenerative diseases; ocular diseases	Renkawek et al. (1994), Dimberg et al. (2008), Kase et al. (2011)
4		HSP70	Lung cancer; acute myeloid; leukemia; colorectal cancer; gastric cancer	Kocsis et al. (2010), Lee et al. (2013), Piszcz et al. (2014)
5		HSP90	Lung adenocarcinoma; esophageal squamous cell carcinoma; breast cancer	Park et al. (2015), Xu et al. (2016), Moyano et al. (2021)
6	Large HSPs	HSP110	Brain ischemia, kainic acid-dependent cell injury, cerebellar Purkinje cell degeneration	Ni and Lee (2007), Sokka et al. (2007), Su and Li (2016)

#### 5.1.1 α-Crystallins

α-crystallins belonging to the sHSP superfamily possess two isoforms: αA-crystallin (HSPB4) and αB-crystallin (HSPB5). They are characterized by the presence of a conserved crystallin domain flanked by a variable N-terminal domain and C-terminal extension. In humans, αA-crystallin is distributed only in the eye lens, while αB-crystallin is found in different tissues (Sprague-Piercy et al., 2021). In the ocular lens, αB-crystallin is predominantly located on the rough ER with smooth membranes (Suetsugu et al., 2010). In the ER stress cell model, a high level of α-crystallins was detected (Ito et al., 2005). It is found that αA-crystallin can recognize misfolded subunits of epithelial Na^+^ channel (ENaC) on the ER membrane and degrade ENaC subunits via the ERAD process (Kashlan et al., 2007). Intriguingly, αB-crystallin negatively regulated apoptosis induced by ER stress (Dou et al., 2012). A study illustrated that a mutant of αB-crystallin named αB-crystallin R120G induced ER stress and impaired Ca^2+^ regulation, resulting in aging-related cardiac dysfunction, arrhythmias, decreased autonomic tone, and shortened lifespan in the mice model (Jiao et al., 2014). In summary, these studies opened new exploratory research avenues with the possibility of identifying α-crystallins as a potential treatment target.

### 5.2 HSP70

HSP70 proteins possess a highly conserved 44 kDa N-terminal ATPase domain, 18 kDa substrate-binding domain, and 10 kDa C-terminal domain (Liu et al., 2012). The expression of HSP70 has been reported to be present in the extracellular milieu via independent of ER-Golgi-mediated protein trafficking to prevent aggregation and promote refolding of misfolded denatured proteins, as well as solubilizing aggregated proteins and cooperating with cellular degradation machinery. Among its functions, HSP70s act as sentinel chaperones, protecting cells against the deleterious effects (Craig, 2018). The member of HSP70s, GRP78 (also known as HSPA5), given that it has been extensively discussed in other parts of this review.

### 5.3 HSP90

HSP90 encoded by gene HSPC1∼5 (Kampinga et al., 2009) is composed of five members in the features of highly conserved molecular chaperones involved in modulating many cellular processes under both physiological and stress conditions. Glucose-regulated protein 94 (GRP-94) is an HSP90 analogue resident in ER (Lamoureux et al., 2014).

### 5.4 HSP110

HSP110, identified in the ER of all eukaryotic cells (Hatayama et al., 1997), is an essential component in almost all eukaryotic cells. HSPH4, also known as 150 kDa oxygen-regulated protein (ORP150), belonging to the HSP110 family, serves as a nucleotide exchange factor for GRP78 in the ER due to its ADP-ATP exchange function (Dragovic et al., 2006) and regulates immunoglobulin folding and assembly in concert with GRP78 (Easton et al., 2000), as well as directly participates ER stress as an independent chaperone (Behnke et al., 2015; Nowakowska et al., 2020). Expression of HSPH4 in the ER at very high levels was induced by brain ischemia (Su and Li, 2016), kainic acid-dependent cell injury (Sokka et al., 2007), and cerebellar Purkinje cell degeneration (Ni and Lee, 2007). However, to date, no drug targeting HSP110 was described.

## 6 Link of Intestinal Disease and ER Stress

Solid evidence has demonstrated that abnormal levels of ER stress are emerging as a driving factor for a wide variety of diseases, including ischemia/reperfusion injury, diabetes, neurodegeneration, and cancer. Environmental stresses, such as microbial infection from food-borne pathogens, are colonized by a diverse and complex community of commensal and pathogenic microorganisms, including bacteria, viruses, and fungi. These can cause intestinal inflammation and systemic stress responses and are also associated with ER stress and devastating major mental disorders. Notably, metabolic studies have shown that these microbes generate metabolites serving as energy sources for cell metabolism, promote the development and the functionality of the immune system, and prevent colonization by pathogenic microorganisms. Different cell types differ in their preferred mode of metabolism to harness the energy and generate its required set of metabolites. As solid cancers grow, ER stress is inevitably induced by hypoxic and nutrient insufficiency. Furthermore, observations illustrated that activation of the UPR in hypoxic tumors contributes to exacerbating autophagy. In conclusion, ER stress plays an important role in many diseases.

### 6.1 IBD and ER Stress

Inflammatory bowel disease (IBD) is a family of chronic inflammatory diseases of all or part of the digestive tract, characterized by a complex and dysregulated immune response. Incidence rates worldwide are increasing. Crohn’s disease (CD) and ulcerative colitis (UC) are the two primary forms of IBD, which has long received attention from scientists interested in a variety of recurrent and unprovoked symptoms appearance; however, most remain unknown.

Recently, an increasing amount of direct experimental evidence indicates that ER stress has been implicated as a major stress-response contributor in the process of occurrence and development of IBD (Rees et al., 2020; Zhang et al., 2021). First of all, various physiological and pathological situations produce alterations in the ER in the animal model of DSS-induced colitis IBD (Yin et al., 2021). This theory was further confirmed in the cells and organoids from human specimens, even the tissue directly derived from IBD patients (Pierre et al., 2020; Rees et al., 2020). Enteroid-derived primary cell monolayers generated from IBD subjects exhibited significantly lower basal expression of GRP78 and higher basal CHOP expression and XBP1s/XBP1u ratios compared to the healthy control subjects. Specifically, ER stress-related genes obtained from the CD patients’ intestinal mucosa, including ATF3, DNAJC3, STC2, DDIT3, CALR, HSPA5, and HSP90B1, showed high expression in comparison with the normal group. Most commonly, changes in mucus associated with Grp78 expression have been increased in the ileal mucosa of CD patients. At the same time, XBP1s levels were also increased between inflamed and non-inflamed ileal/colonic CD or colonic UC mucosa. At the same time, the expression of ERN1 genes is upregulated to support the increased demands of XBP1s protein from autophagy activity. Although these studies are not identical in a significant change of mucosal inflammation in patients with CD and UC, these data indicated that IRE1 activity was increased in the CD ileum and colon (Kaser et al., 2008). The results were also supported by recent discoveries. In the ileum and the colon of patients with CD, immunochemistry confirmed the presence of ER stress in the mucosa of ileal and colonic ulcer edges (Pierre et al., 2020). It is worth mentioning that ER stress may contribute to the development of fibrosis in patients with CD (Li et al., 2020). The normal human intestinal fibroblast cell line CCD-18Co cells incubated supernatant of HT-29 cells pre-conditioned by TM occurred the fibroblast to myofibroblast differentiation (Vieujean et al., 2021). Of note, these ER stress response proteins are also involved in restoring the integrity of the epithelial barrier through participating in proper protein folding, ERAD, or ER stress-mediated apoptosis.

Importantly, an increasing number of studies indicate considerable overlap of IBD pathophysiology and IRE1. Ern1 knockout (KO) in mice directly leads to death during the state of embryonic process (Iwawaki et al., 2009), while Ern2 KO in mice results in increased ER stress, shortened latency of colitis, exacerbated DSS-induced colitis, induced idiopathic colitis (Bertolotti et al., 2001; Kurashima and Kiyono, 2017), and aggravated severity of colitis. Different results were drawn in the case of genetic ablation of Ire1α in IECs. Conditional deletion of IRE1α in IECs results in loss of goblet cells through induction of CHOP and failure of intestinal epithelial barrier function. This contributes to sensitize cells to LPS, an endotoxin from bacteria, eventually leading to spontaneous colitis and higher susceptibility to chemical-induced colitis in mice (Zhang et al., 2015). These results suggest that to maintain intestinal epithelial homeostasis, the IRE1α functions play an important role in defending against IBD. Reports have revealed that the human IRE1β gene maps to the pericentromeric region of chromosome 16 within a single PAC interval of the polymorphic simple sequence repeat D16s417 harboring one or more genes implicated in modifying the risk for the development of IBD (Hugot et al., 1996; Brant et al., 1998; Wang et al., 1998). The role of IRE1β can be uncovered by the deletion of genes. IRE1β^–/–^ mice exhibited highly elevated BiP in the colonic mucosa. Through a longitudinal follow-up of up to 1 year, IRE1β^–/–^ mice did not spontaneously develop intestinal inflammation, when exposed to DSS, and IRE1β^–/–^ mice pronounced earlier alterations compared to wild-type mice, including marked shortening of the long axis, inflammatory cells infiltration consisting predominantly of mononuclear cells, and ulceration consisting of focal lesions interspersed with areas with intact surface epithelium, which suggested that mutant mice were prone to developed colitis earlier. Apart from this, expression of ICAM-1, an inflammation marker, was earlier detected in mutant mice than that in wild type (Bertolotti et al., 2001).

In order to elucidate the role of the Xbp1 in the intestine, Kaser et al. (2008) generated the model of Xbp1^flox/flox^ Villin-Cre (Xbp1^−/−^) transgenic mice. Through this, Kaser et al. demonstrated that the model mice were characterized by boosting responses of IEC to mucosal inflammatory signals including JNK, CXCL1, and TNFα, exhibiting abscess and ulceration in the intestinal crypt and multinucleated infiltration in the lamina propria. These data suggested that Xbp1^−/−^ mice had more severe intestinal inflammation and higher susceptibility to spontaneous enteritis than wild-type mice (Adolph et al., 2013).

More recently, studies have linked ATF6 to IBD. Expression of activated ATF6, TNF, and other inflammatory cytokines increased in ileal IECs from CD patients and in organoids from Xbp1^ΔIEC^ mice (Stengel et al., 2020).

Mucins build the first defense line, protecting the gastrointestinal tract from antimicrobial factors to maintain homeostasis. Goblet cells serve as mucin-producing cells found scattered among other cells of the intestinal villi and crypts in lesser numbers than the absorptive cells. ER stress increased MUC5AC protein production and secretion in human airway epithelial cells via the IRE1α and XBP1 signaling pathways (Xu et al., 2021). More interestingly, studies indicated that cells devoted to secreting encompassing goblet and Paneth cells in the gut are the major targets of intestinal ER stress (Zhao et al., 2010; Kaser et al., 2011; Larabi et al., 2020). In Xbp1^−/−^ mice, Paneth cells were almost undetectable in the intestine tract by electron microscopy and analyzing mRNA. Alterations also took place in the number and size of goblet cells in the intestine, not in the colon upon Xbp1 deletion, despite the slight decrease in numbers and size (Kaser et al., 2008). The Winnie and Eeyore mouse model with MUC2 missense mutations showed elevated ER stress of goblet cells, which decreased the number of goblet cells, impaired mucus barrier, and developed colitis spontaneously.

Notably, goblet cell and secreted mucus phenotypes represent important differences between the two diseases: Crohn’s disease is characterized by an increase in goblet cells and a thicker mucus layer (Dvorak et al., 1980; Trabucchi et al., 1986). In contrast, there is a reduction in goblet cells, decrease in MUC2 production (Tytgat et al., 1996; Van Klinken et al., 1999; Wang et al., 2019), accumulation of MUC2 precursor (Hanski et al., 1999), and reduction in secreted mucus in UC (Heazlewood et al., 2008; Samoila et al., 2020). These studies highlight the sensitivity of goblet cells to ER stress and the importance of proper MUC2 processing in maintaining goblet cell health and overall mucosal homeostasis.

More recently, it was suggested that the degree of drug-induced ERS aggravation correlates with MUC2 expression levels and that high mucin-producing cells are more susceptible to drug-mediated ERS aggravation (Dilly et al., 2020).

Collectively, ER stress exerts its multifaceted role on IBD.

### 6.2 Colon Cancer

Many studies have established the complex and important roles of ER stress in contributing to the development and progression of colorectal cancer (Ryan et al., 2016). In this review, we summarized the suggestions and proposed new therapeutic strategies based on the results of the present and previous studies. In explant tissue from mucinous colon cancers, significantly high expression levels of MUC2, GRP78, ATF4, and CHOP protein were detected (Dilly et al., 2020).

Notably, PERK has an explicit connection with the tumor cells’ survival. PERK phosphorylation of eIF2α was required to grow solid tumors (Bi et al., 2005). Cells sharing the ability to resist ER stress and multiple chemotherapeutic agents revealed higher PERK expression through a genetic profile analysis. In turn, inducible silencing PERK reduced tumor growth and restored chemotherapeutic sensitivity in resistant tumor xenografts (Salaroglio et al., 2017). Angiogenesis is generated to escape the predicament of hypoxic conditions in cancer formation. Strikingly, the PERK-ATF4 pathway can directly promote vascular endothelial growth factor (VEGF) and suppress angiogenesis inhibitors such as angiostatin, PF4, and matrix metalloproteinase (MMP) (Blais et al., 2006; Wang et al., 2012).

IRE1α is also a vital participant in tumor development. As research reported, deletion of IRE1α decreased VEGF, which is crucial for angiogenesis and inhibits tumor growth (Drogat et al., 2007). Another report proposed that the knockdown of IRE1α suppressed the proliferation of colon cancer cells *in vitro* and xenograft growth *in vivo* through repressing the expression of β-catenin, a key factor that drives colonic tumorigenesis, thereby activating eIF2α signaling. The IRE1a-specific inhibitor 4μ8C could suppress the production of β-catenin, inhibit the proliferation of colon cancer cells, repress colon CSCs, and prevent xenograft growth. The results suggest that IRE1α has a critical role in colonic tumorigenesis, and IRE1α targeting might be a strategy for treating colon cancers (Li et al., 2017). Another study showed that, in the case of gene deletion of IRE1α, colon cancer cells are sensitive to mitogen-activated protein kinase (MEK) inhibitor KRAS mutants (Sustic et al., 2018). However, the deletion of IRE1α resulted in tumor invasion (Auf et al., 2010).

Compared to the control group, the expression levels of IRE1β and MUC2 proteins were decreased in the tumor and resisted inflammation, thus promoting the occurrence and development of colonic tumors (Dai et al., 2019). IRE1β appears to specifically play its RNase role in highly differentiated secretory cells such as goblet cells, thereby degrading the mRNA encoding specific secretory proteins, including MUC2 (Nakamura et al., 2011; Tsuru et al., 2013). Colorectal cancer (CRC) tissue samples were surgically resected tumor tissues from patients from 44 to 82 years with colorectal adenocarcinoma. IRE1β and MUC2, the mRNA expression and protein levels, were significantly decreased compared to the noncancerous tissues. Strikingly, it was identified that the IRE1β mRNA expression levels were positively associated with the MUC2 mRNA expression levels. IRE1β, regardless of the transcriptional and translational level, was significantly higher in those patients with lymphatic metastasis or stages III-IV of CRC (Jiang et al., 2017).

Meanwhile, the role of the IRE1-XBP1 pathway in the tumor has been comprehensively explored (Bi et al., 2005; Feldman et al., 2005). XBP1s can partly protect the tumor from hypoxia, while deficiency of *Xbp1* can hinder hypoxic tumor growth (Romero-Ramirez et al., 2004; Feldman et al., 2005). Simultaneously, apoptosis was increased in the Xbp1-deficient cells, thereby inhibiting tumor growth. Taken together, these studies directly suggested that XBP1 is an essential survival factor for hypoxic stress and tumor growth. Four colon cancer cell lines (DLD1, SW480, HCT15, and WiDr) showed increased expression of XBP1. The tendency of high expression of XBP1 in the level of mRNA and protein also occurred in the patients who suffered from colorectal adenoma and adenocarcinoma (Fujimoto et al., 2007).

ER stress destroys the effectiveness of traditional treatments, including chemotherapy, hormone therapy, and targeted therapies in the course of cancer treatment (Avril et al., 2017), decreases the risk for recurrence, and accelerates death (Andruska et al., 2015). In other words, ER stress contributes to tumor survival, especially in colorectal cancer (CRC) (Ryan et al., 2016). ER stress can alleviate cancer cells death and promote survival and metastasis. One study utilized the colon tissue of Winnie that is a mouse model of chronic ER stress and wild-type BLK6 mice to validate the expression of survivin, one of the IAP inhibitors of apoptosis proteins (IAPs) that binds to caspase activation involved in apoptosis to protect cells from apoptosis. The results showed that the level of survivin in Winnie is higher than that in the wild type. Results indicated that, with the presence of ER stress, the level of ER stress markers and anti-inflammatory cytokine IL-10 is remarkably higher. In contrast, the level of inflammatory markers is significantly lower compared to those with the absence of ER stress, which directly served as a convincing argument demonstrating the beneficial role of ER stress in the survival of cancer cells. As Nrf2 involved in chemoresistance is promoted by PERK in HT29 colon cancer cells (Salaroglio et al., 2017), researchers attached the importance of PERK and CHOP to GADD34 dephosphorylating eIF2α, providing an insight that suppression of the CHOP-GADD34 axis may be a tumor survival mechanism (Clarke et al., 2014). Notably, the induction of GADD34 may have a broad effect on tumor-suppressive effects. In contrast, the sensibility of human colon cancer cells to 5-fluorouracil (5-FU) chemotherapy would recover after genetic or pharmacologic inhibition of the PERK-ATF4 pathway (Shi et al., 2019).

However, when ER stress is severe and induces cell death, divert results will work out. Many studies in the cell level targeted for colon cancer treatment pointed out that cell death induced by ER stress may target antitumor medicine (Tung et al., 2015; Dilly et al., 2020; Zhang et al., 2021).

Tremendous studies have corroborated the vital roles of GRP78 in the occurrence of colon cancer, resulting from GRP78 working as the main chaperone participating in protein folding. Firstly, the authors artificially separated GRP78 positive and negative sub-populations from HM7 and GRP78 HCT116 cell lines using anti-GRP78 antibody-coated magnetic beads and elucidated that receptor-positive and receptor-negative tumor cells manifest different properties in colorectal cancer. GRP78 negative cells were characterized by highly proliferative and significant growth in tumor size and bigger metastases than GRP78 positive cells. What is more, after silencing GRP78 expression using siRNA oligomers in the positive sub-population, proliferation induced a 53% increase. On the contrary, cells pre-incubated with doxorubicin, which induced GRP78 high expression, exhibited reduced proliferation and tumor growth (Hardy et al., 2012).

Studies have reported that autophagy induced by chemicals including A23187, TM, TN, and brefeldin A only conferred protection in specific colon cancer cells such as HCT116 cell lines rather than all the colon cancer cells (Ding et al., 2007). Parallel to the protective role of autophagy for a colon cancer cell, studies have corroborated the autophagic inhibition effect on anti-colon cancer via apoptosis induced by p53 activation and ER stress *in vivo* and *in vitro*. Inhibiting autophagy, specifically in the epithelial cell, was achieved in *Atg5* ^flox/flox^/K19^CreERT+^. Treating the mutant and controlling mice (*Atg5* ^flox/flox^) with azoxymethane/dextran sodium sulfate (AOM/DSS) induced colon cancer. A significant difference existed in the aspect of maximum and total sizes of the tumor due to smaller size in Atg5-deficient mice. Meanwhile, increased expression levels of cleaved PARP, cleaved caspase-3, and GRP78 were detected in mutant mice, indicating that apoptosis was elicited and autophagic inhibition had an antitumor effect (Sakitani et al., 2015).

Colon cancer stem cells were characterized by high Wnt pathway activity and chlorogenic potential. They were more resistant to chemotherapy, whereas the features disappeared when the cells became more differentiated. Intriguingly, ER stress was reported to have differentiating effects conserved between normal intestinal stem cells and colon cancer stem cells. Subtilase cytotoxin AB, a bacterium-derived protease that explicitly cleaves ER chaperone GRP78, was administrated to the colon cancer stem cells to activate ER stress *in vitro*. When colon cancer stem cells with a high Wnt pathway sorted by FACS were exposed to subtilize cytotoxin AB or TG, stem cell markers such as OLFM4 and LGR5 were lost. On the contrary, differentiated cell makers increased. The expression of the inhibitor of intestinal stem cell-cycle progression P21Cip1/Waf1 or cyclin-dependent kinase inhibitor 1 (CDKN1) was upregulated, and enterocyte markers CK20, VIL2, SI, and FABP2 increased. Of note, MUC2, a hallmark of secretory goblet cells, was downregulated, arguing that ER stress was subscribed to induce intestinal epithelial stem cells differentiation toward an absorptive phenotype rather than a secretory phenotype. It is worth noting that transient UPR activation may promote regenerating these cells, given that the expression of LGR5 was increased after exposure to subtilase cytotoxin AB for 48 h. Colon cancer stem cells are suggested to be more resistant to conventional chemotherapy compared to differentiated cancer cells (Colak et al., 2014). UPR activation sensitized cells toward oxaliplatin and 5-FU resulting from high expression of caspase-3. To a great extent, the *in vivo* manifestations were observed. Transient UPR activation elicited by treating mice with salubrinal, an inhibitor of phosphatizing of eIF2α, resulted in increased growth of xenografts derived from colon cancer stem cells. The above results indicated that UPR activation provides a window of opportunity to improve chemotherapy outcomes (Wielenga et al., 2015).

To date, many reports have linked ER stress to 5-FU resistance. 5-FU-resistant SNUC5 colon cancer cells (SNUC5/FUR cells) and drug-sensitive SNUC5 cells were used to explore the consequence of arising resistant reaction. SNUC5/FUR cells exhibited higher expression of GRP78, XBP1s, ATF6, phosphorylated PERK, and eIF2ɑ and recovered sensitivity to 5-FU when transfected with siRNA against GRP78, ATF6, and ERK (Kim et al., 2016). However, a recent study provided evidence that apoptosis of SNUC5/FUR cells can be induced by ER stress (Piao et al., 2021).

## 7 Treatment of Intestinal Diseases Targeting ER Stress

The therapeutic applications and intriguing pharmacological properties of ER stress have attracted great attention from medical researchers. Plenty of new studies corroborated that tauroursodeoxycholic acid (TUDCA) has an important role in protecting against ER stress, and treatment with TUDCA ameliorates various models of colitis in mice (Cao et al., 2013; Hino et al., 2014; Laukens et al., 2014). Colon-targeted prodrugs of 4-PBA were designed and synthesized, and their colon-targeting property was evaluated. Furthermore, the drug’s effectiveness was shown by regulating ER stress in a model of colitis in rats (Kim et al., 2020).

In addition, many ER stress inhibitors are generated, such as benzodiazepines, baicalein, and 1-deoxymannojirimycin hydrochloride (Farooqi et al., 2015). More recently, surprisingly, a study disclosed that *Bifidobacterium* and *Lactobacillus acidophilus* suppressed ER-mediated goblet cell stress and reduced colonic inflammation (Kim et al., 2019; Engevik et al., 2021).

In particular, small molecule modulators of the ER stress target normal binding sites by activating sites of three sensors machinery and altering the function of IRE1α’s RNase (Papandreou et al., 2011; Volkmann et al., 2011; Cross et al., 2012; Mimura et al., 2012). Several studies have found evidence, direct or indirect, to support the importance of IRE1α’s kinase in multiple pathway-involved in the regulation of the cell differentiation and proliferation of the host antitumor response in colon cancer. First, salicylaldehyde analogs were the first reported systematically in the field, such as specific IRE1 RNase inhibitors and 3-methoxy-6-bromosalicylaldehyde that selectively represses XBP-1, and mRNAs is, in turn, a selective degradation by IRE1 (Volkmann et al., 2011). In addition, salicylaldehyde-based inhibitors have many strengths, including SFT-083010, MKC-3946, and 4u8c, which contain ideal reactive electrophile for binding the sites of IRE1’s RNase via a covalent bond to form a functional pharmacophore, similar to the geometry of the reduced form of the Schiff base of salicylaldehyde in the K907 can active site of the RNase (Cross et al., 2012; Sanches et al., 2014), and the kinase and RNase activity of IRE1α are regulated by kinase inhibitors through two different modes. One is to occupy the kinase ATP-binding site of IRE1α that activates Xbp1 mRNA splicing, contributing to adaptive UPR and ER homeostasis. The other is to inhibit RNase through the same ATP binding sites. Consequently, ATP-competitive inhibitors bind and stabilize can cause allosteric switching of IRE1α′s RNase either on or off.

Apart from the above, highly potent and selective inhibitors of PERK’s kinase domain become hotspots for selective ER stress. Accordingly, the inhibition of PERK, GSK2606414, and GSK2656157 has emerged as a promising cancer therapy (Atkins et al., 2013), including the ATP-competitive inhibitor. However, we recognize that these studies are not exhaustive; these examples of small-molecule inhibitors against IRE1a and PERK highlight the therapeutic potential and risks of targeting the UPR in cancer. A more thorough discussion of therapeutics against the UPR should be referred to (Maly and Papa, 2014; Hetz et al., 2019). Due to the remarkably increased tendency of chaperone induced by ER-stress response, many studies have targeted chaperone as a therapeutic target. GRP94, as a common chaperone, HSP90 inhibitor geldanamycin and AUY922 causes cell death (Marcu et al., 2002; Wang et al., 2018). Strikingly, some of the agents are nonspecific inhibitors with lower toxicity displaying power and selectivity for GRP94 (Duerfeldt et al., 2012). See the summaries in Table 2.

**TABLE 2:** List of drugs inhibiting ER stress.

1	Drug name	Mechanism of inhibition	References
2	4-Phenylbutyric acid (4-PBA)	Suppressing oxidative stress by attenuating ER stress	Kim et al. (2020), Choi et al. (2021)
3	Tauroursodeoxycholic acid (TUDCA)	Stabilizing unfolded proteins to prevent their aggregation from the induction of autophagy	Ozcan et al. (2006)
4	Benzodiazepines	Selectively suppressing cell death associated with the IRE1 pathway though modulating phosphorylation of ASK1 and inhibiting downstream activation of JNK and p38 MAPK.	Kim et al. (2009), Zou et al. (2015)
5	Baicalein	Attenuating pyroptosis, alleviating ER stress-mediated apoptosis to activate the autophagy process	Wu et al. (2020)
6	1-Deoxymannojirimycin	Inhibiting N-glycan processing in the ER to attenuate ER stress-induced cell death	Lu et al. (2006), Miyake and Nagai (2009)
7	Toyocamycin	IRE1 RNase inhibitors	Chien et al. (2014)
8	3-Ethoxy-5,6-dibromosa-licylaldehyde	A salicylaldehyde analog, IRE1 RNase inhibitors	Volkmann et al. (2011), Chien et al. (2014)
		A non-competitive inhibitor with respect to the XBP-1 RNA substrate	
9	SFT-083010	Aldehyde-based covalent as the inhibitors of the IRE1α′s RNase	Papandreou et al. (2011), Mimura et al. (2012)
10	MKC-3946	Inhibiting IRE1α′s RNase	Mimura et al. (2012), Zhang et al. (2014), Sun et al. (2016)
11	4u8c	Inhibiting IRE1α′s RNase	Nelson et al. (2018), Wang et al. (2019a), Cao et al. (2019)
12	GSK2606414	Inhibiting PERK’s kinase	Axten et al. (2013)
13	GSK2656157	An ATP-competitive inhibitor of PERK enzyme	Axten et al. (2013)
14	Geldanamycin	Inhibiting GRP 94	Marcu et al. (2002)
		Nonspecific HSP 90 inhibitor	
15	17-AAG (Phases I–III), SNX-5422 (Phase I), CNF 2024 (Phase II), and NVP-AUY922 (Phases I/II)	Specific HSP 90 inhibitor	Duerfeldt et al. (2012)
16	Overexpression of BiP1 or BiP3	Impede the accumulation of unfolded or misfolded proteins in the ER	Otero et al. (2010)

## 8 Conclusion

ER stress has been hotly debated due to its vital role in disease initiation and progression. What role does ER stress act in the process of disease? Is it beneficial, harmful, or both? Much research was attempted to unravel the mysterious veil of ER stress. ER stress, as a reaction to the unbalanced environment, is protective for cells. However, things can turn upside down when stress is chronic or otherwise irremediable. Therefore, future research should tackle this challenging question to understand the intricate interaction between ER stress and diseases. How ER stress can continue to play its positive role is another issue. Many studies focused on the treatment targeting ER stress by inhibiting or activating some ER stress members, which is a potent way to alleviate diseases. So far, there has been limited research that explores the relationship between stress and disease. Therefore, more effort in this area would further our understanding and treatment of diseases.

## Abbreviations

DDIT3, DNA damage-inducible transcript 3; DTT, dithiothreitol; eIF2α, eukaryotic translation initiation factor 2α; ERO1α, endoplasmic reticulum oxidoreductin 1; GADD34, growth arrest and DNA damage-inducible 34; GRP94, glucose-regulated protein 94; HSP, heat shock protein; IKK, IκB kinase; MAMs, mitochondria-associated membranes; MAPKs, mitogen-activated protein kinases; Mfn2, mitofusin-2; NLRP3, nod-like receptor protein-3; PP1C, protein phosphatase 1C; RIDD, regulated IRE1-dependent mRNA decay; ROS, reactive oxygen species; S1P, site-1 protease; S2P, site-2 protease; TM, tunicamycin; TRAIL-R1/DR4, TNF-related apoptosis-inducing ligand (TRAIL) receptor 1; TRAIL-R2/DR5, TNF-related apoptosis-inducing ligand (TRAIL) receptor 2; TUDCA, tauroursodeoxycholic acid; TXNIP, thioredoxin interacting protein; VEGF, vascular endothelial growth factor; XBP1, X-binding protein 1; XIAP, X-linked inhibitor of apoptosis protein.

## References

[B1] Adolph T. E. Tomczak M. F. Niederreiter L. Ko H.-J. Böck J. Martinez-Naves E. (2013). Paneth Cells as a Site of Origin for Intestinal Inflammation. Nature 503, 272–276. 10.1038/nature12599 24089213 PMC3862182

[B2] Alderson T. R. Ying J. Bax A. Benesch J. L. P. Baldwin A. J. (2020). Conditional Disorder in Small Heat-Shock Proteins. J. Mol. Biol. 432, 3033–3049. 10.1016/j.jmb.2020.02.003 32081587 PMC7245567

[B3] Andruska N. D. Zheng X. Yang X. Mao C. Cherian M. M. Mahapatra L. (2015). Estrogen Receptor α Inhibitor Activates the Unfolded Protein Response, Blocks Protein Synthesis, and Induces Tumor Regression. Proc. Natl. Acad. Sci. USA 112, 4737–4742. 10.1073/pnas.1403685112 25825714 PMC4403155

[B4] Arrigo A. P. (1990). Tumor Necrosis Factor Induces the Rapid Phosphorylation of the Mammalian Heat Shock Protein Hsp28. Mol. Cel Biol 10, 1276–1280. 10.1128/mcb.10.3.1276-1280.1990 PMC361020 2304467

[B5] Atkins C. Liu Q. Minthorn E. Zhang S.-Y. Figueroa D. J. Moss K. (2013). Characterization of a Novel PERK Kinase Inhibitor with Antitumor and Antiangiogenic Activity. Cancer Res. 73, 1993–2002. 10.1158/0008-5472.can-12-3109 23333938

[B6] Auf G. Jabouille A. Guerit S. Pineau R. Delugin M. Bouchecareilh M. (2010). Inositol-requiring Enzyme 1 Is a Key Regulator of Angiogenesis and Invasion in Malignant Glioma. Proc. Natl. Acad. Sci. 107, 15553–15558. 10.1073/pnas.0914072107 20702765 PMC2932600

[B7] Avril T. Vauléon E. Chevet E. (2017). Endoplasmic Reticulum Stress Signaling and Chemotherapy Resistance in Solid Cancers. Oncogenesis 6, e373. 10.1038/oncsis.2017.72 28846078 PMC5608920

[B8] Axten J. M. Romeril S. P. Shu A. Ralph J. Medina J. R. Feng Y. (2013). Discovery of GSK2656157: An Optimized PERK Inhibitor Selected for Preclinical Development. ACS Med. Chem. Lett. 4, 964–968. 10.1021/ml400228e 24900593 PMC4027568

[B9] B'Chir W. Maurin A. C. Carraro V. Averous J. Jousse C. Muranishi Y. (2013). The eIF2α/ATF4 Pathway Is Essential for Stress-Induced Autophagy Gene Expression. Nucleic Acids Res. 41, 7683–7699. 10.1093/nar/gkt563 23804767 PMC3763548

[B10] Behnke J. Feige M. J. Hendershot L. M. (2015). BiP and its Nucleotide Exchange Factors Grp170 and Sil1: Mechanisms of Action and Biological Functions. J. Mol. Biol. 427, 1589–1608. 10.1016/j.jmb.2015.02.011 25698114 PMC4356644

[B11] Bertolotti A. Wang X. Novoa I. Jungreis R. Schlessinger K. Cho J. H. (2001). Increased Sensitivity to Dextran Sodium Sulfate Colitis in IRE1β-Deficient Mice. J. Clin. Invest. 107, 585–593. 10.1172/jci11476 11238559 PMC199427

[B12] Bertolotti A. Zhang Y. Hendershot L. M. Harding H. P. Ron D. (2000). Dynamic Interaction of BiP and ER Stress Transducers in the Unfolded-Protein Response. Nat. Cel Biol 2, 326–332. 10.1038/35014014 10854322

[B13] Bettigole S. E. Glimcher L. H. (2015). Endoplasmic Reticulum Stress in Immunity. Annu. Rev. Immunol. 33, 107–138. 10.1146/annurev-immunol-032414-112116 25493331

[B14] Bi M. Naczki C. Koritzinsky M. Fels D. Blais J. Hu N. (2005). ER Stress-Regulated Translation Increases Tolerance to Extreme Hypoxia and Promotes Tumor Growth. Embo J. 24, 3470–3481. 10.1038/sj.emboj.7600777 16148948 PMC1276162

[B15] Blais J. D. Addison C. L. Edge R. Falls T. Zhao H. Wary K. (2006). Perk-dependent Translational Regulation Promotes Tumor Cell Adaptation and Angiogenesis in Response to Hypoxic Stress. Mol. Cel Biol 26, 9517–9532. 10.1128/mcb.01145-06 PMC1698539 17030613

[B16] Brant S. Fu Y. Fields C. Baltazar R. Ravenhill G. Pickles M. (1998). American Families with Crohn's Disease Have strong Evidence for Linkage to Chromosome 16 but Not Chromosome 12. Gastroenterology 115, 1056–1061. 10.1016/s0016-5085(98)70073-3 9797357

[B17] Brennan M. A. Cookson B. T. (2000). Salmonella Induces Macrophage Death by Caspase-1-dependent Necrosis. Mol. Microbiol. 38, 31–40. 10.1046/j.1365-2958.2000.02103.x 11029688

[B18] Campbell J. M. Crenshaw J. D. Polo J. (2013). The Biological Stress of Early Weaned Piglets. J. Anim. Sci Biotechnol 4, 19. 10.1186/2049-1891-4-19 23631414 PMC3651348

[B19] Cao S. S. Zimmermann E. M. Chuang B. M. Song B. Nwokoye A. Wilkinson J. E. (2013). The Unfolded Protein Response and Chemical Chaperones Reduce Protein Misfolding and Colitis in Mice. Gastroenterology 144, 989–1000. 10.1053/j.gastro.2013.01.023 23336977 PMC3751190

[B20] Cao X. He Y. Li X. Xu Y. Liu X. (2019). The IRE1α-XBP1 Pathway Function in Hypoxia-Induced Pulmonary Vascular Remodeling, Is Upregulated by Quercetin, Inhibits Apoptosis and Partially Reverses the Effect of Quercetin in PASMCs. Am. J. Transl Res. 11, 641–654. 30899368 PMC6413268

[B21] Carreras-Sureda A. Pihán P. Hetz C. (2017). The Unfolded Protein Response: At the Intersection between Endoplasmic Reticulum Function and Mitochondrial Bioenergetics. Front. Oncol. 7, 55. 10.3389/fonc.2017.00055 28421160 PMC5377016

[B22] Caspers G.-J. Leunissen J. A. M. de Jong W. W. (1995). The Expanding Small Heat-Shock Protein Family, and Structure Predictions of the Conserved "α-Crystallin Domain". J. Mol. Evol. 40, 238–248. 10.1007/bf00163229 7723051

[B23] Chan S. W. Egan P. A. (2005). Hepatitis C Virus Envelope Proteins Regulate CHOP via Induction of the Unfolded Protein Response. FASEB j. 19, 1510–1512. 10.1096/fj.04-3455fje 16006626

[B24] Chen X. Shen J. Prywes R. (2002). The Luminal Domain of ATF6 Senses Endoplasmic Reticulum (ER) Stress and Causes Translocation of ATF6 from the ER to the Golgi. J. Biol. Chem. 277, 13045–13052. 10.1074/jbc.m110636200 11821395

[B25] Cheng S.-B. Nakashima A. Huber W. J. Davis S. Banerjee S. Huang Z. (2019). Pyroptosis Is a Critical Inflammatory Pathway in the Placenta from Early Onset Preeclampsia and in Human Trophoblasts Exposed to Hypoxia and Endoplasmic Reticulum Stressors. Cell Death Dis 10, 927. 10.1038/s41419-019-2162-4 31804457 PMC6895177

[B26] Chiang W.-C. Hiramatsu N. Messah C. Kroeger H. Lin J. H. (2012). Selective Activation of ATF6 and PERK Endoplasmic Reticulum Stress Signaling Pathways Prevent Mutant Rhodopsin Accumulation. Invest. Ophthalmol. Vis. Sci. 53, 7159–7166. 10.1167/iovs.12-10222 22956602 PMC3474590

[B27] Chien W. Ding L.-W. Sun Q.-Y. Torres-Fernandez L. A. Tan S. Z. Xiao J. (2014). Selective Inhibition of Unfolded Protein Response Induces Apoptosis in Pancreatic Cancer Cells. Oncotarget 5, 4881–4894. 10.18632/oncotarget.2051 24952679 PMC4148107

[B28] Choi A. Y. Choi J. H. Lee J. Y. Yoon K. S. Choe W. Ha J. (2010). Apigenin Protects HT22 Murine Hippocampal Neuronal Cells Against Endoplasmic Reticulum Stress-Induced Apoptosis. Neurochem. Int. 57, 143–152. 10.1016/j.neuint.2010.05.006 20493918

[B29] Choi Y. Lee E. G. Jeong J. H. Yoo W. H. (2021). 4-Phenylbutyric Acid, a Potent Endoplasmic Reticulum Stress Inhibitor, Attenuates the Severity of Collagen-Induced Arthritis in Mice via Inhibition of Proliferation and Inflammatory Responses of Synovial Fibroblasts. Kaohsiung J. Med. Sci. 10.1002/kjm2.12376 PMC11896533 33759334

[B30] Chrispeels M. J. Greenwood J. S. (1987). Heat Stress Enhances Phytohemagglutinin Synthesis but Inhibits its Transport Out of the Endoplasmic Reticulum. Plant Physiol. 83, 778–784. 10.1104/pp.83.4.778 16665338 PMC1056449

[B31] Clarke H. J. Chambers J. E. Liniker E. Marciniak S. J. (2014). Endoplasmic Reticulum Stress in Malignancy. Cancer cell 25, 563–573. 10.1016/j.ccr.2014.03.015 24823636

[B32] Colak S. Zimberlin C. D. Fessler E. Hogdal L. Prasetyanti P. R. Grandela C. M. (2014). Decreased Mitochondrial Priming Determines Chemoresistance of colon Cancer Stem Cells. Cell Death Differ 21, 1170–1177. 10.1038/cdd.2014.37 24682005 PMC4207483

[B33] Collins P. L. Hightower L. E. (1982). Newcastle Disease Virus Stimulates the Cellular Accumulation of Stress (Heat Shock) mRNAs and Proteins. J. Virol. 44, 703–707. 10.1128/jvi.44.2.703-707.1982 7143579 PMC256315

[B34] Cox J. S. Shamu C. E. Walter P. (1993). Transcriptional Induction of Genes Encoding Endoplasmic Reticulum Resident Proteins Requires a Transmembrane Protein Kinase. Cell 73, 1197–1206. 10.1016/0092-8674(93)90648-a 8513503

[B35] Craig E. A. (2018). Hsp70 at the Membrane: Driving Protein Translocation. BMC Biol. 16, 11. 10.1186/s12915-017-0474-3 29343244 PMC5773037

[B36] Craig E. A. Schlesinger M. J. (1985). The Heat Shock Respons. Crit. Rev. Biochem. 18, 239–280. 10.3109/10409238509085135 2412760

[B37] Crompton M. Virji S. Doyle V. Johnson N. Ward J. M. (1999). The Mitochondrial Permeability Transition Pore. Biochem. Soc. Symp. 66 (Pt 2), 167–179. 10.1042/bss0660167 10989666

[B38] Cross B. C. S. Bond P. J. Sadowski P. G. Jha B. K. Zak J. Goodman J. M. (2012). The Molecular Basis for Selective Inhibition of Unconventional mRNA Splicing by an IRE1-Binding Small Molecule. Proc. Natl. Acad. Sci. 109, E869–E878. 10.1073/pnas.1115623109 22315414 PMC3326519

[B39] Dai F. Dong S. Rong Z. Xuan Q. Chen P. Chen M. (2019). Expression of Inositol-Requiring Enzyme 1β Is Downregulated in Azoxymethane/dextran Sulfate Sodium-Induced Mouse Colonic Tumors. Exp. Ther. Med. 17, 3181–3188. 10.3892/etm.2019.7317 30936991 PMC6434252

[B40] de la Peña de Torres E. (1978). Effects of P,p'-DDT on the Parenchima of Liver in Treated NMRI Mice: II. Ultrastructural Changes (Author's Transl). Arch. Farmacol Toxicol. 4, 339–348. 747411

[B41] Dilly A. K. Honick B. D. Lee Y. J. Bartlett D. L. Choudry H. A. (2020). Synergistic Apoptosis Following Endoplasmic Reticulum Stress Aggravation in Mucinous colon Cancer. Orphanet J. Rare Dis. 15, 211. 10.1186/s13023-020-01499-1 32811515 PMC7437176

[B42] Dimberg A. Rylova S. Dieterich L. C. Olsson A.-K. Schiller P. Wikner C. (2008). αB-crystallin Promotes Tumor Angiogenesis by Increasing Vascular Survival during Tube Morphogenesis. Blood 111, 2015–2023. 10.1182/blood-2007-04-087841 18063749

[B43] Ding W.-X. Ni H.-M. Gao W. Hou Y.-F. Melan M. A. Chen X. (2007). Differential Effects of Endoplasmic Reticulum Stress-Induced Autophagy on Cell Survival. J. Biol. Chem. 282, 4702–4710. 10.1074/jbc.m609267200 17135238

[B44] Dong T. Liao D. Liu X. Lei X. (2015). Using Small Molecules to Dissect Non-apoptotic Programmed Cell Death: Necroptosis, Ferroptosis, and Pyroptosis. Chembiochem 16, 2557–2561. 10.1002/cbic.201500422 26388514

[B45] Dou G. Sreekumar P. G. Spee C. He S. Ryan S. J. Kannan R. (2012). Deficiency of αB Crystallin Augments ER Stress-Induced Apoptosis by Enhancing Mitochondrial Dysfunction. Free Radic. Biol. Med. 53, 1111–1122. 10.1016/j.freeradbiomed.2012.06.042 22781655 PMC3454510

[B46] Dragovic Z. Broadley S. A. Shomura Y. Bracher A. Hartl F. U. (2006). Molecular Chaperones of the Hsp110 Family Act as Nucleotide Exchange Factors of Hsp70s. Embo J. 25, 2519–2528. 10.1038/sj.emboj.7601138 16688212 PMC1478182

[B47] Drogat B. Auguste P. Nguyen D. T. Bouchecareilh M. Pineau R. Nalbantoglu J. (2007). IRE1 Signaling Is Essential for Ischemia-Induced Vascular Endothelial Growth Factor-A Expression and Contributes to Angiogenesis and Tumor Growth *In Vivo* . Cancer Res. 67, 6700–6707. 10.1158/0008-5472.can-06-3235 17638880

[B48] Duerfeldt A. S. Peterson L. B. Maynard J. C. Ng C. L. Eletto D. Ostrovsky O. (2012). Development of a Grp94 Inhibitor. J. Am. Chem. Soc. 134, 9796–9804. 10.1021/ja303477g 22642269 PMC3414055

[B49] Dvorak A. M. Osage J. E. Monahan R. A. Dickersin G. R. (1980). Crohn's Disease: Transmission Electron Microscopic Studies. Hum. Pathol. 11, 620–634. 10.1016/s0046-8177(80)80073-6 6161074

[B50] Easton D. P. Kaneko Y. Subjeck J. R. (2000). The Hsp110 and Grp170 Stress Proteins: Newly Recognized Relatives of the Hsp70s. Cell Stress Chaper 5, 276–290. 10.1379/1466-1268(2000)005<0276:thagsp>2.0.co;2 PMC312858 11048651

[B51] Emelyanov V. V. (2002). Phylogenetic Relationships of Organellar Hsp90 Homologs Reveal Fundamental Differences to Organellar Hsp70 and Hsp60 Evolution. Gene 299, 125–133. 10.1016/s0378-1119(02)01021-1 12459260

[B52] Engevik M. A. Herrmann B. Ruan W. Engevik A. C. Engevik K. A. Ihekweazu F. (2021). Bifidobacterium Dentium-Derived Y-Glutamylcysteine Suppresses ER-Mediated Goblet Cell Stress and Reduces TNBS-Driven Colonic Inflammation. Gut Microbes 13, 1–21. 10.1080/19490976.2021.1902717 PMC8128206 33985416

[B53] Fan Y. Simmen T. (2019). Mechanistic Connections between Endoplasmic Reticulum (ER) Redox Control and Mitochondrial Metabolism. Cells 8. 10.3390/cells8091071 PMC6769559 31547228

[B54] Farooqi A. A. Li K.-T. Fayyaz S. Chang Y.-T. Ismail M. Liaw C.-C. (2015). Anticancer Drugs for the Modulation of Endoplasmic Reticulum Stress and Oxidative Stress. Tumor Biol. 36, 5743–5752. 10.1007/s13277-015-3797-0 PMC4546701 26188905

[B55] Feldman D. E. Chauhan V. Koong A. C. (2005). The Unfolded Protein Response: a Novel Component of the Hypoxic Stress Response in Tumors. Mol. Cancer Res. 3, 597–605. 10.1158/1541-7786.mcr-05-0221 16317085

[B56] Fujimoto T. Yoshimatsu K. Watanabe K. Yokomizo H. Otani T. Matsumoto A. (2007). Overexpression of Human X-Box Binding Protein 1 (XBP-1) in Colorectal Adenomas and Adenocarcinomas. Anticancer Res. 27, 127–131. 17352224

[B57] Gething M.-J. Sambrook J. (1992). Protein Folding in the Cell. Nature 355, 33–45. 10.1038/355033a0 1731198

[B58] Haas I. G. Wabl M. (1983). Immunoglobulin Heavy Chain Binding Protein. Nature 306, 387–389. 10.1038/306387a0 6417546

[B59] Hamman B. D. Hendershot L. M. Johnson A. E. (1998). BiP Maintains the Permeability Barrier of the ER Membrane by Sealing the Lumenal End of the Translocon Pore before and Early in Translocation. Cell 92, 747–758. 10.1016/s0092-8674(00)81403-8 9529251

[B60] Han J. Back S. H. Hur J. Lin Y.-H. Gildersleeve R. Shan J. (2013). ER-stress-induced Transcriptional Regulation Increases Protein Synthesis Leading to Cell Death. Nat. Cel Biol 15, 481–490. 10.1038/ncb2738 PMC3692270 23624402

[B61] Hanski C. Born M. Foss H. D. Marowski B. Mansmann U. Arasth K. (1999). Defective post-transcriptional Processing of MUC2 Mucin in Ulcerative Colitis and in Crohn's Disease Increases Detectability of the MUC2 Protein Core. J. Pathol. 188, 304–311. 10.1002/(sici)1096-9896(199907)188:3<304:aid-path375>3.0.co;2-a 10419600

[B62] Harding H. P. Novoa I. Zhang Y. Zeng H. Wek R. Schapira M. (2000a). Regulated Translation Initiation Controls Stress-Induced Gene Expression in Mammalian Cells. Mol. Cel. 6, 1099–1108. 10.1016/s1097-2765(00)00108-8 11106749

[B63] Harding H. P. Zhang Y. Bertolotti A. Zeng H. Ron D. (2000b). Perk Is Essential for Translational Regulation and Cell Survival during the Unfolded Protein Response. Mol. Cel. 5, 897–904. 10.1016/s1097-2765(00)80330-5 10882126

[B64] Harding H. P. Zhang Y. Ron D. (1999). Protein Translation and Folding Are Coupled by an Endoplasmic-Reticulum-Resident Kinase. Nature 397, 271–274. 10.1038/16729 9930704

[B65] Harding H. P. Zhang Y. Zeng H. Novoa I. Lu P. D. Calfon M. (2003). An Integrated Stress Response Regulates Amino Acid Metabolism and Resistance to Oxidative Stress. Mol. Cel. 11, 619–633. 10.1016/s1097-2765(03)00105-9 12667446

[B66] Hardy B. Raiter A. Yakimov M. Vilkin A. Niv Y. (2012). Colon Cancer Cells Expressing Cell Surface GRP78 as a Marker for Reduced Tumorigenicity. Cell Oncol. 35, 345–354. 10.1007/s13402-012-0094-4 PMC12995008 22945507

[B67] Hassler J. Cao S. S. Kaufman R. J. (2012). IRE1, a Double-Edged Sword in Pre-miRNA Slicing and Cell Death. Developmental Cel. 23, 921–923. 10.1016/j.devcel.2012.10.025 PMC3684431 23153490

[B68] Hatayama T. Takigawa T. Takeuchi S. Shiota K. (1997). Characteristic Expression of High Molecular Mass Heat Shock Protein HSP105 during Mouse Embryo Development. Cell Struct. Funct. 22, 517–525. 10.1247/csf.22.517 9431456

[B69] Heazlewood C. K. Cook M. C. Eri R. Price G. R. Tauro S. B. Taupin D. (2008). Aberrant Mucin Assembly in Mice Causes Endoplasmic Reticulum Stress and Spontaneous Inflammation Resembling Ulcerative Colitis. Plos Med. 5, e54. 10.1371/journal.pmed.0050054 18318598 PMC2270292

[B70] Hetz C. Axten J. M. Patterson J. B. (2019). Pharmacological Targeting of the Unfolded Protein Response for Disease Intervention. Nat. Chem. Biol. 15, 764–775. 10.1038/s41589-019-0326-2 31320759

[B71] Hetz C. Bernasconi P. Fisher J. Lee A.-H. Bassik M. C. Antonsson B. (2006). Proapoptotic BAX and BAK Modulate the Unfolded Protein Response by a Direct Interaction with IRE1α. Science 312, 572–576. 10.1126/science.1123480 16645094

[B72] Hetz C. (2012). The Unfolded Protein Response: Controlling Cell Fate Decisions under ER Stress and beyond. Nat. Rev. Mol. Cel Biol 13, 89–102. 10.1038/nrm3270 22251901

[B73] Hino K. Saito A. Asada R. Kanemoto S. Imaizumi K. (2014). Increased Susceptibility to Dextran Sulfate Sodium-Induced Colitis in the Endoplasmic Reticulum Stress Transducer OASIS Deficient Mice. PLoS One 9, e88048. 10.1371/journal.pone.0088048 24498426 PMC3912207

[B74] Hollien J. Lin J. H. Li H. Stevens N. Walter P. Weissman J. S. (2009). Regulated Ire1-dependent Decay of Messenger RNAs in Mammalian Cells. J. Cel. Biol. 186, 323–331. 10.1083/jcb.200903014 PMC2728407 19651891

[B75] Hollien J. Weissman J. S. (2006). Decay of Endoplasmic Reticulum-Localized mRNAs during the Unfolded Protein Response. Science 313, 104–107. 10.1126/science.1129631 16825573

[B76] Hong M. Luo S. Baumeister P. Huang J.-M. Gogia R. K. Li M. (2004). Underglycosylation of ATF6 as a Novel Sensing Mechanism for Activation of the Unfolded Protein Response. J. Biol. Chem. 279, 11354–11363. 10.1074/jbc.m309804200 14699159

[B77] Hu P. Han Z. Couvillon A. D. Kaufman R. J. Exton J. H. (2006). Autocrine Tumor Necrosis Factor Alpha Links Endoplasmic Reticulum Stress to the Membrane Death Receptor Pathway through IRE1α-Mediated NF-Κb Activation and Down-Regulation of TRAF2 Expression. Mol. Cel Biol 26, 3071–3084. 10.1128/mcb.26.8.3071-3084.2006 PMC1446932 16581782

[B78] Hu X. Deng J. Yu T. Chen S. Ge Y. Zhou Z. (2019). ATF4 Deficiency Promotes Intestinal Inflammation in Mice by Reducing Uptake of Glutamine and Expression of Antimicrobial Peptides. Gastroenterology 156, 1098–1111. 10.1053/j.gastro.2018.11.033 30452920

[B79] Huang J. Wan L. Lu H. Li X. (2018). High Expression of Active ATF6 Aggravates Endoplasmic Reticulum Stress-induced V-ascular E-ndothelial C-ell A-poptosis through the M-itochondrial A-poptotic P-athway. Mol. Med. Rep. 17, 6483–6489. 10.3892/mmr.2018.8658 29512699 PMC5928631

[B80] Hugot J.-P. Laurent-Puig P. Gower-Rousseau C. Olson J. M. Lee J. C. Beaugerie L. (1996). Mapping of a Susceptibility Locus for Crohn's Disease on Chromosome 16. Nature 379, 821–823. 10.1038/379821a0 8587604

[B81] Inaguma Y. Goto S. Shinohara H. Hasegawa K. Ohshima K. Kato K. (1993). Physiological and Pathological Changes in Levels of the Two Small Stress Proteins, HSP27 and ∝B Crystallin, in Rat Hindlimb Muscles1. J. Biochem. 114, 378–384. 10.1093/oxfordjournals.jbchem.a124184 8282729

[B82] Inoue Y. Moriyasu Y. (2006). Degradation of Membrane Phospholipids in Plant Cells Cultured in Sucrose-free Medium. Autophagy 2, 244–246. 10.4161/auto.2745 16874091

[B83] Ip Y. T. Davis R. J. (1998). Signal Transduction by the C-Jun N-Terminal Kinase (JNK) - from Inflammation to Development. Curr. Opin. Cel. Biol. 10, 205–219. 10.1016/s0955-0674(98)80143-9 9561845

[B84] Ishikawa T. Kashima M. Nagano A. J. Ishikawa-Fujiwara T. Kamei Y. Todo T. (2017). Unfolded Protein Response Transducer IRE1-Mediated Signaling Independent of XBP1 mRNA Splicing Is Not Required for Growth and Development of Medaka Fish. Elife 6. 10.7554/eLife.26845 PMC5636610 28952924

[B85] Ito H. Iwamoto I. Inaguma Y. Takizawa T. Nagata K.-i. Asano T. (2005). Endoplasmic Reticulum Stress Induces the Phosphorylation of Small Heat Shock Protein, Hsp27. J. Cel. Biochem. 95, 932–941. 10.1002/jcb.20445 15864808

[B86] Iurlaro R. Muñoz-Pinedo C. (2016). Cell Death Induced by Endoplasmic Reticulum Stress. Febs J. 283, 2640–2652. 10.1111/febs.13598 26587781

[B87] Iwawaki T. Akai R. Yamanaka S. Kohno K. (2009). Function of IRE1 Alpha in the Placenta Is Essential for Placental Development and Embryonic Viability. Proc. Natl. Acad. Sci. 106, 16657–16662. 10.1073/pnas.0903775106 19805353 PMC2757843

[B88] Iwawaki T. Hosoda A. Okuda T. Kamigori Y. Nomura-Furuwatari C. Kimata Y. (2001). Translational Control by the ER Transmembrane Kinase/ribonuclease IRE1 under ER Stress. Nat. Cel Biol 3, 158–164. 10.1038/35055065 11175748

[B89] Jiang Y. Zhou Y. Zheng Y. Guo H. Gao L. Chen P. (2017). Expression of Inositol-Requiring Enzyme 1β Is Downregulated in Colorectal Cancer. Oncol. Lett. 13, 1109–1118. 10.3892/ol.2017.5590 28454221 PMC5403352

[B90] Jiao Q. Sanbe A. Zhang X. Liu J.-P. Minamisawa S. (2014). αB-Crystallin R120G Variant Causes Cardiac Arrhythmias and Alterations in the Expression of Ca2+-Handling Proteins and Endoplasmic Reticulum Stress in Mice. Clin. Exp. Pharmacol. Physiol. 41, 589–599. 10.1111/1440-1681.12253 24825000

[B91] Johnston J. A. Ward C. L. Kopito R. R. (1998). Aggresomes: a Cellular Response to Misfolded Proteins. J. Cel. Biol. 143, 1883–1898. 10.1083/jcb.143.7.1883 PMC2175217 9864362

[B92] Jorgensen I. Lopez J. P. Laufer S. A. Miao E. A. (2016). IL-1β, IL-18, and Eicosanoids Promote Neutrophil Recruitment to Pore-Induced Intracellular Traps Following Pyroptosis. Eur. J. Immunol. 46, 2761–2766. 10.1002/eji.201646647 27682622 PMC5138142

[B93] Jovcevski B. Kelly M. A. Rote A. P. Berg T. Gastall H. Y. Benesch J. L. P. (2015). Phosphomimics Destabilize Hsp27 Oligomeric Assemblies and Enhance Chaperone Activity. Chem. Biol. 22, 186–195. 10.1016/j.chembiol.2015.01.001 25699602

[B94] Kampinga H. H. Hageman J. Vos M. J. Kubota H. Tanguay R. M. Bruford E. A. (2009). Guidelines for the Nomenclature of the Human Heat Shock Proteins. Cell Stress and Chaperones 14, 105–111. 10.1007/s12192-008-0068-7 18663603 PMC2673902

[B95] Kase S. Ishida S. Rao N. A. (2011). Increased Expression of αA-crystallin in Human Diabetic Eye. Int. J. Mol. Med. 28, 505–511. 10.3892/ijmm.2011.708 21617844

[B96] Kaser A. Blumberg R. S. (2010). Endoplasmic Reticulum Stress and Intestinal Inflammation. Mucosal Immunol. 3, 11–16. 10.1038/mi.2009.122 19865077 PMC4592136

[B97] Kaser A. Lee A.-H. Franke A. Glickman J. N. Zeissig S. Tilg H. (2008). XBP1 Links ER Stress to Intestinal Inflammation and Confers Genetic Risk for Human Inflammatory Bowel Disease. Cell 134, 743–756. 10.1016/j.cell.2008.07.021 18775308 PMC2586148

[B98] Kaser A. Tomczak M. Blumberg R. S. (2011). "ER Stress(ed Out)!": Paneth Cells and Ischemia-Reperfusion Injury of the Small Intestine. Gastroenterology 140, 393–396. 10.1053/j.gastro.2010.12.015 21172333 PMC4594951

[B99] Kashlan O. B. Mueller G. M. Qamar M. Z. Poland P. A. Ahner A. Rubenstein R. C. (2007). Small Heat Shock Protein αA-crystallin Regulates Epithelial Sodium Channel Expression. J. Biol. Chem. 282, 28149–28156. 10.1074/jbc.m703409200 17664274 PMC2361386

[B100] Ke R. Wang Y. Hong S. Xiao L. (2020). Endoplasmic Reticulum Stress Related Factor IRE1α Regulates TXNIP/NLRP3-mediated Pyroptosis in Diabetic Nephropathy. Exp. Cel. Res. 396, 112293. 10.1016/j.yexcr.2020.112293 32950473

[B101] Kennedy D. Mnich K. Oommen D. Chakravarthy R. Almeida-Souza L. Krols M. (2017). HSPB1 Facilitates ERK-Mediated Phosphorylation and Degradation of BIM to Attenuate Endoplasmic Reticulum Stress-Induced Apoptosis. Cel Death Dis 8, e3026. 10.1038/cddis.2017.408 PMC5596589 29048431

[B102] Kikuchi D. Tanimoto K. Nakayama K. (2016). CREB Is Activated by ER Stress and Modulates the Unfolded Protein Response by Regulating the Expression of IRE1α and PERK. Biochem. Biophysical Res. Commun. 469, 243–250. 10.1016/j.bbrc.2015.11.113 26642955

[B103] Kim D. H. Kim S. Lee J. H. Kim J. H. Che X. Ma H. W. (2019). Lactobacillus Acidophilus Suppresses Intestinal Inflammation by Inhibiting Endoplasmic Reticulum Stress. J. Gastroenterol. Hepatol. 34, 178–185. 10.1111/jgh.14362 29933526

[B104] Kim I. Shu C.-W. Xu W. Shiau C.-W. Grant D. Vasile S. (2009). Chemical Biology Investigation of Cell Death Pathways Activated by Endoplasmic Reticulum Stress Reveals Cytoprotective Modulators of ASK1. J. Biol. Chem. 284, 1593–1603. 10.1074/jbc.m807308200 19004820 PMC2615512

[B105] Kim I. Xu W. Reed J. C. (2008). Cell Death and Endoplasmic Reticulum Stress: Disease Relevance and Therapeutic Opportunities. Nat. Rev. Drug Discov. 7, 1013–1030. 10.1038/nrd2755 19043451

[B106] Kim J. K. Kang K. A. Piao M. J. Ryu Y. S. Han X. Fernando P. M. D. J. (2016). Endoplasmic Reticulum Stress Induces 5-fluorouracil Resistance in Human colon Cancer Cells. Environ. Toxicol. Pharmacol. 44, 128–133. 10.1016/j.etap.2016.05.005 27163731

[B107] Kim S. Lee S. Lee H. Ju S. Park S. Kwon D. (2020). A Colon-Targeted Prodrug, 4-Phenylbutyric Acid-Glutamic Acid Conjugate, Ameliorates 2,4-Dinitrobenzenesulfonic Acid-Induced Colitis in Rats. Pharmaceutics 12. 10.3390/pharmaceutics12090843 PMC7558321 32899177

[B108] Klemenz R. Fröhli E. Aoyama A. Hoffmann S. Simpson R. J. Moritz R. L. (1991). Alpha B Crystallin Accumulation Is a Specific Response to Ha-Ras and V-Mos Oncogene Expression in Mouse NIH 3T3 Fibroblasts. Mol. Cel. Biol. 11, 803–812. 10.1128/mcb.11.2.803 PMC359732 1846673

[B109] Koch G. Smith M. Macer D. Webster P. Mortara R. (1986). Endoplasmic Reticulum Contains a Common, Abundant Calcium-Binding Glycoprotein, Endoplasmin. J. Cel. Sci. 86, 217–232. 10.1242/jcs.86.1.217 3308928

[B110] Kocsis J. Madaras B. Tóth É. K. Füst G. Prohászka Z. (2010). Serum Level of Soluble 70-kD Heat Shock Protein Is Associated with High Mortality in Patients with Colorectal Cancer without Distant Metastasis. Cell Stress and Chaperones 15, 143–151. 10.1007/s12192-009-0128-7 19578980 PMC2866989

[B111] Kokame K. Kato H. Miyata T. (2001). Identification of ERSE-II, a New Cis-Acting Element Responsible for the ATF6-dependent Mammalian Unfolded Protein Response. J. Biol. Chem. 276, 9199–9205. 10.1074/jbc.m010486200 11112790

[B112] Kouroku Y. Fujita E. Tanida I. Ueno T. Isoai A. Kumagai H. (2007). ER Stress (PERK/eIF2α Phosphorylation) Mediates the Polyglutamine-Induced LC3 Conversion, an Essential Step for Autophagy Formation. Cel Death Differ 14, 230–239. 10.1038/sj.cdd.4401984 16794605

[B113] Krajewski S. Tanaka S. Takayama S. Schibler M. J. Fenton W. Reed J. C. (1993). Investigation of the Subcellular Distribution of the Bcl-2 Oncoprotein: Residence in the Nuclear Envelope, Endoplasmic Reticulum, and Outer Mitochondrial Membranes. Cancer Res. 53, 4701–4714. 8402648

[B114] Krishna P. Gloor G. (2001). &cestflwr; The Hsp90 Family of Proteins in *Arabidopsis thaliana* . Cell Stress Chaper 6, 238–246. 10.1379/1466-1268(2001)006<0238:thfopi>2.0.co;2 PMC434405 11599565

[B115] Kurashima Y. Kiyono H. (2017). Mucosal Ecological Network of Epithelium and Immune Cells for Gut Homeostasis and Tissue Healing. Annu. Rev. Immunol. 35, 119–147. 10.1146/annurev-immunol-051116-052424 28125357

[B116] Lai E. Teodoro T. Volchuk A. (2007). Endoplasmic Reticulum Stress: Signaling the Unfolded Protein Response. Physiology 22, 193–201. 10.1152/physiol.00050.2006 17557940

[B117] Lajoie P. Snapp E. L. (2020). Size‐dependent Secretory Protein Reflux into the Cytosol in Association with Acute Endoplasmic Reticulum Stress. Traffic 21, 419–429. 10.1111/tra.12729 32246734 PMC7317852

[B118] Lamoureux F. Thomas C. Yin M.-J. Fazli L. Zoubeidi A. Gleave M. E. (2014). Suppression of Heat Shock Protein 27 Using OGX-427 Induces Endoplasmic Reticulum Stress and Potentiates Heat Shock Protein 90 Inhibitors to Delay Castrate-Resistant Prostate Cancer. Eur. Urol. 66, 145–155. 10.1016/j.eururo.2013.12.019 24411988 PMC4079118

[B119] Larabi A. Barnich N. Nguyen H. T. T. (2020). New Insights into the Interplay between Autophagy, Gut Microbiota and Inflammatory Responses in IBD. Autophagy 16, 38–51. 10.1080/15548627.2019.1635384 31286804 PMC6984609

[B120] Laukens D. Devisscher L. Van den Bossche L. Hindryckx P. Vandenbroucke R. E. Vandewynckel Y.-P. (2014). Tauroursodeoxycholic Acid Inhibits Experimental Colitis by Preventing Early Intestinal Epithelial Cell Death. Lab. Invest. 94, 1419–1430. 10.1038/labinvest.2014.117 25310532

[B121] Lee A.-H. Iwakoshi N. N. Anderson K. C. Glimcher L. H. (2003a). Proteasome Inhibitors Disrupt the Unfolded Protein Response in Myeloma Cells. Proc. Natl. Acad. Sci. 100, 9946–9951. 10.1073/pnas.1334037100 12902539 PMC187896

[B122] Lee A.-H. Iwakoshi N. N. Glimcher L. H. (2003b). XBP-1 Regulates a Subset of Endoplasmic Reticulum Resident Chaperone Genes in the Unfolded Protein Response. Mol. Cel Biol 23, 7448–7459. 10.1128/mcb.23.21.7448-7459.2003 PMC207643 14559994

[B123] Lee A. S. Bell J. Ting J. (1984). Biochemical Characterization of the 94- and 78-kilodalton Glucose-Regulated Proteins in Hamster Fibroblasts. J. Biol. Chem. 259, 4616–4621. 10.1016/s0021-9258(17)43091-2 6707023

[B124] Lee H. W. Lee E. H. Kim S.-H. Roh M. S. Jung S. B. Choi Y. C. (2013). Heat Shock Protein 70 (HSP70) Expression Is Associated with Poor Prognosis in Intestinal Type Gastric Cancer. Virchows Arch. 463, 489–495. 10.1007/s00428-013-1461-x 23913168

[B125] Li C. Grider J. R. Murthy K. S. Bohl J. Rivet E. Wieghard N. (2020). Endoplasmic Reticulum Stress in Subepithelial Myofibroblasts Increases the TGF-Β1 Activity that Regulates Fibrosis in Crohn's Disease. Inflamm. Bowel Dis. 26, 809–819. 10.1093/ibd/izaa015 32031621 PMC7324000

[B126] Li G. Mongillo M. Chin K.-T. Harding H. Ron D. Marks A. R. (2009). Role of ERO1-α-Mediated Stimulation of Inositol 1,4,5-triphosphate Receptor Activity in Endoplasmic Reticulum Stress-Induced Apoptosis. J. Cel. Biol. 186, 783–792. 10.1083/jcb.200904060 PMC2753154 19752026

[B127] Li J. Wang J. J. Zhang S. X. (2011). Preconditioning with Endoplasmic Reticulum Stress Mitigates Retinal Endothelial Inflammation via Activation of X-Box Binding Protein 1. J. Biol. Chem. 286, 4912–4921. 10.1074/jbc.m110.199729 21138840 PMC3039327

[B128] Li X.-X. Zhang H.-S. Xu Y.-M. Zhang R.-J. Chen Y. Fan L. (2017). Knockdown of IRE1α Inhibits Colonic Tumorigenesis through Decreasing β-catenin and IRE1α Targeting Suppresses colon Cancer Cells. Oncogene 36, 6738–6746. 10.1038/onc.2017.284 28825721

[B129] Lin J. H. Li H. Yasumura D. Cohen H. R. Zhang C. Panning B. (2007). IRE1 Signaling Affects Cell Fate during the Unfolded Protein Response. Science 318, 944–949. 10.1126/science.1146361 17991856 PMC3670588

[B130] Lisbona F. Rojas-Rivera D. Thielen P. Zamorano S. Todd D. Martinon F. (2009). BAX Inhibitor-1 Is a Negative Regulator of the ER Stress Sensor IRE1α. Mol. Cel 33, 679–691. 10.1016/j.molcel.2009.02.017 PMC2818874 19328063

[B131] Liu J.-X. Howell S. H. (2010). Endoplasmic Reticulum Protein Quality Control and its Relationship to Environmental Stress Responses in Plants. Plant Cell 22, 2930–2942. 10.1105/tpc.110.078154 20876830 PMC2965551

[B132] Liu J.-X. Srivastava R. Che P. Howell S. H. (2007). Salt Stress Responses in Arabidopsis Utilize a Signal Transduction Pathway Related to Endoplasmic Reticulum Stress Signaling. Plant J. 51, 897–909. 10.1111/j.1365-313x.2007.03195.x 17662035 PMC2156172

[B133] Liu T. Daniels C. K. Cao S. (2012). Comprehensive Review on the HSC70 Functions, Interactions with Related Molecules and Involvement in Clinical Diseases and Therapeutic Potential. Pharmacol. Ther. 136, 354–374. 10.1016/j.pharmthera.2012.08.014 22960394

[B134] Liu Y. Bassham D. C. (2012). Autophagy: Pathways for Self-Eating in Plant Cells. Annu. Rev. Plant Biol. 63, 215–237. 10.1146/annurev-arplant-042811-105441 22242963

[B135] Liu Y. László C. Liu Y. Liu W. Chen X. Evans S. C. (2010). Regulation of G1 Arrest and Apoptosis in Hypoxia by PERK and GCN2-Mediated eIF2α Phosphorylation. Neoplasia 12, 61–IN6. 10.1593/neo.91354 20072654 PMC2805884

[B136] Logue S. E. Cleary P. Saveljeva S. Samali A. (2013). New Directions in ER Stress-Induced Cell Death. Apoptosis 18, 537–546. 10.1007/s10495-013-0818-6 23430059

[B137] Lu M. Lawrence D. A. Marsters S. Acosta-Alvear D. Kimmig P. Mendez A. S. (2014). Opposing Unfolded-Protein-Response Signals Converge on Death Receptor 5 to Control Apoptosis. Science 345, 98–101. 10.1126/science.1254312 24994655 PMC4284148

[B138] Lu P. D. Harding H. P. Ron D. (2004). Translation Reinitiation at Alternative Open reading Frames Regulates Gene Expression in an Integrated Stress Response. J. Cel. Biol. 167, 27–33. 10.1083/jcb.200408003 PMC2172506 15479734

[B139] Lu Y. Xu Y.-Y. Fan K.-Y. Shen Z.-H. (2006). 1-Deoxymannojirimycin, the α1,2-mannosidase Inhibitor, Induced Cellular Endoplasmic Reticulum Stress in Human Hepatocarcinoma Cell 7721. Biochem. biophysical Res. Commun. 344, 221–225. 10.1016/j.bbrc.2006.03.111 16615997

[B140] Luo B. Li B. Wang W. Liu X. Xia Y. Zhang C. (2014). NLRP3 Gene Silencing Ameliorates Diabetic Cardiomyopathy in a Type 2 Diabetes Rat Model. PloS one 9, e104771. 10.1371/journal.pone.0104771 25136835 PMC4138036

[B141] Ma Y. Hendershot L. M. (2002). &cestchinlong;The Mammalian Endoplasmic Reticulum as a Sensor for Cellular Stress. Cell Stress Chaper 7, 222–229. 10.1379/1466-1268(2002)007<0222:tmeraa>2.0.co;2 PMC514821 12380691

[B142] Malhotra J. D. Kaufman R. J. (2007). Endoplasmic Reticulum Stress and Oxidative Stress: a Vicious Cycle or a Double-Edged Sword? Antioxid. Redox Signaling 9, 2277–2294. 10.1089/ars.2007.1782 17979528

[B143] Maly D. J. Papa F. R. (2014). Druggable Sensors of the Unfolded Protein Response. Nat. Chem. Biol. 10, 892–901. 10.1038/nchembio.1664 25325700 PMC4664160

[B144] Man S. M. Karki R. Kanneganti T.-D. (2017). Molecular Mechanisms and Functions of Pyroptosis, Inflammatory Caspases and Inflammasomes in Infectious Diseases. Immunol. Rev. 277, 61–75. 10.1111/imr.12534 28462526 PMC5416822

[B145] Marciniak S. J. Yun C. Y. Oyadomari S. Novoa I. Zhang Y. Jungreis R. (2004). CHOP Induces Death by Promoting Protein Synthesis and Oxidation in the Stressed Endoplasmic Reticulum. Genes Dev. 18, 3066–3077. 10.1101/gad.1250704 15601821 PMC535917

[B146] Marcu M. G. Doyle M. Bertolotti A. Ron D. Hendershot L. Neckers L. (2002). Heat Shock Protein 90 Modulates the Unfolded Protein Response by Stabilizing IRE1α. Mol. Cel Biol 22, 8506–8513. 10.1128/mcb.22.24.8506-8513.2002 PMC139892 12446770

[B147] McCullough K. D. Martindale J. L. Klotz L.-O. Aw T.-Y. Holbrook N. J. (2001). Gadd153 Sensitizes Cells to Endoplasmic Reticulum Stress by Down-Regulating Bcl2 and Perturbing the Cellular Redox State. Mol. Cel Biol 21, 1249–1259. 10.1128/mcb.21.4.1249-1259.2001 PMC99578 11158311

[B148] Meyerovich K. Ortis F. Allagnat F. Cardozo A. K. (2016). Endoplasmic Reticulum Stress and the Unfolded Protein Response in Pancreatic Islet Inflammation. J. Mol. Endocrinol. 57, R1–R17. 10.1530/jme-15-0306 27067637

[B149] Mimura N. Fulciniti M. Gorgun G. Tai Y.-T. Cirstea D. Santo L. (2012). Blockade of XBP1 Splicing by Inhibition of IRE1α Is a Promising Therapeutic Option in Multiple Myeloma. Blood 119, 5772–5781. 10.1182/blood-2011-07-366633 22538852 PMC3382937

[B150] Miyake K. Nagai K. (2009). Inhibition of α-mannosidase Attenuates Endoplasmic Reticulum Stress-Induced Neuronal Cell Death. Neurotoxicology 30, 144–150. 10.1016/j.neuro.2008.10.010 19028522

[B151] Mizushima N. (2005). The Pleiotropic Role of Autophagy: from Protein Metabolism to Bactericide. Cel Death Differ 12 (Suppl. 2), 1535–1541. 10.1038/sj.cdd.4401728 16247501

[B152] Mkrtchian S. Baryshev M. Sargsyan E. Chatzistamou I. Volakaki A.-A. Chaviaras N. (2008). ERp29, an Endoplasmic Reticulum Secretion Factor Is Involved in the Growth of Breast Tumor Xenografts. Mol. Carcinog. 47, 886–892. 10.1002/mc.20444 18395818

[B153] Mori K. (2000). Tripartite Management of Unfolded Proteins in the Endoplasmic Reticulum. Cell 101, 451–454. 10.1016/s0092-8674(00)80855-7 10850487

[B154] Mori-Iwamoto S. Kuramitsu Y. Ryozawa S. Mikuria K. Fujimoto M. Maehara S.-I. (2007). Proteomics Finding Heat Shock Protein 27 as a Biomarker for Resistance of Pancreatic Cancer Cells to Gemcitabine. Int. J. Oncol. 31, 1345–1350. 10.3892/ijo.31.6.1345 17982661

[B155] Morimoto R. I. (2008). Proteotoxic Stress and Inducible Chaperone Networks in Neurodegenerative Disease and Aging. Genes Dev. 22, 1427–1438. 10.1101/gad.1657108 18519635 PMC2732416

[B156] Morishima N. Nakanishi K. Takenouchi H. Shibata T. Yasuhiko Y. (2002). An Endoplasmic Reticulum Stress-specific Caspase Cascade in Apoptosis. J. Biol. Chem. 277, 34287–34294. 10.1074/jbc.m204973200 12097332

[B157] Moyano P. Garcia J. M. García J. Pelayo A. Muñoz-Calero P. Frejo M. T. (2021). Aryl Hydrocarbon Receptor Activation Produces Heat Shock Protein 90 and 70 Overexpression, Prostaglandin E2/Wnt/β-Catenin Signaling Disruption, and Cell Proliferation in MCF-7 and MDA-MB-231 Cells after 24 H and 14 Days of Chlorpyrifos Treatment. Chem. Res. Toxicol. 34, 2019–2023. 10.1021/acs.chemrestox.1c00258 34424684 PMC9132385

[B158] Muaddi H. Majumder M. Peidis P. Papadakis A. I. Holcik M. Scheuner D. (2010). Phosphorylation of eIF2α at Serine 51 Is an Important Determinant of Cell Survival and Adaptation to Glucose Deficiency. MBoC 21, 3220–3231. 10.1091/mbc.e10-01-0023 20660158 PMC2938387

[B159] Muñoz J. P. Ivanova S. Sánchez-Wandelmer J. Martínez-Cristóbal P. Noguera E. Sancho A. (2013). Mfn2 Modulates the UPR and Mitochondrial Function via Repression of PERK. EMBO J. 32, 2348–2361. 10.1038/emboj.2013.168 23921556 PMC3770335

[B160] Nadanaka S. Yoshida H. Kano F. Murata M. Mori K. (2004). Activation of Mammalian Unfolded Protein Response Is Compatible with the Quality Control System Operating in the Endoplasmic Reticulum. MBoC 15, 2537–2548. 10.1091/mbc.e03-09-0693 15020717 PMC420080

[B161] Nakagawa T. Yuan J. (2000). Cross-Talk between Two Cysteine Protease Families. J. Cel. Biol. 150, 887–894. 10.1083/jcb.150.4.887 PMC2175271 10953012

[B162] Nakamura D. Tsuru A. Ikegami K. Imagawa Y. Fujimoto N. Kohno K. (2011). Mammalian ER Stress Sensor IRE1β Specifically Down-Regulates the Synthesis of Secretory Pathway Proteins. FEBS Lett. 585, 133–138. 10.1016/j.febslet.2010.12.002 21146530

[B163] Nelson A. M. Carew N. T. Smith S. M. Milcarek C. (2018). RNA Splicing in the Transition from B Cells to Antibody-Secreting Cells: The Influences of ELL2, Small Nuclear RNA, and Endoplasmic Reticulum Stress. J.I. 201, 3073–3083. 10.4049/jimmunol.1800557 PMC6219926 30297340

[B164] Ng D. T. Hiebert S. W. Lamb R. A. (1990). Different Roles of Individual N-Linked Oligosaccharide Chains in Folding, Assembly, and Transport of the Simian Virus 5 Hemagglutinin-Neuraminidase. Mol. Cel Biol 10, 1989–2001. 10.1128/mcb.10.5.1989-2001.1990 PMC360545 2183015

[B165] Ni M. Lee A. S. (2007). ER Chaperones in Mammalian Development and Human Diseases. FEBS Lett. 581, 3641–3651. 10.1016/j.febslet.2007.04.045 17481612 PMC2040386

[B166] Novoa I. Zeng H. Harding H. P. Ron D. (2001). Feedback Inhibition of the Unfolded Protein Response by GADD34-Mediated Dephosphorylation of eIF2α. J. Cel. Biol. 153, 1011–1022. 10.1083/jcb.153.5.1011 PMC2174339 11381086

[B167] Nowakowska M. Gualtieri F. von Rüden E.-L. Hansmann F. Baumgärtner W. Tipold A. (2020). Profiling the Expression of Endoplasmic Reticulum Stress Associated Heat Shock Proteins in Animal Epilepsy Models. Neuroscience 429, 156–172. 10.1016/j.neuroscience.2019.12.015 31887356

[B168] Ogata M. Hino S.-i. Saito A. Morikawa K. Kondo S. Kanemoto S. (2006). Autophagy Is Activated for Cell Survival after Endoplasmic ReticulumStress. Mol. Cel Biol 26, 9220–9231. 10.1128/mcb.01453-06 PMC1698520 17030611

[B169] Ohoka N. Yoshii S. Hattori T. Onozaki K. Hayashi H. (2005). TRB3, a Novel ER Stress-Inducible Gene, Is Induced via ATF4-CHOP Pathway and Is Involved in Cell Death. EMBO J. 24, 1243–1255. 10.1038/sj.emboj.7600596 15775988 PMC556400

[B170] Otero J. H. Lizák B. Hendershot L. M. (2010). Life and Death of a BiP Substrate. Semin. Cel Developmental Biol. 21, 472–478. 10.1016/j.semcdb.2009.12.008 PMC2883687 20026282

[B171] Ozcan U. Yilmaz E. Ozcan L. Furuhashi M. Vaillancourt E. Smith R. O. (2006). Chemical Chaperones Reduce ER Stress and Restore Glucose Homeostasis in a Mouse Model of Type 2 Diabetes. Science 313, 1137–1140. 10.1126/science.1128294 16931765 PMC4741373

[B172] Pain V. M. (1996). Initiation of Protein Synthesis in Eukaryotic Cells. Eur. J. Biochem. 236, 747–771. 10.1111/j.1432-1033.1996.00747.x 8665893

[B173] Papandreou I. Denko N. C. Olson M. Van Melckebeke H. Lust S. Tam A. (2011). Identification of an Ire1alpha Endonuclease Specific Inhibitor with Cytotoxic Activity against Human Multiple Myeloma. Blood 117, 1311–1314. 10.1182/blood-2010-08-303099 21081713 PMC3056474

[B174] Park K.-W. Eun Kim G. Morales R. Moda F. Moreno-Gonzalez I. Concha-Marambio L. (2017). The Endoplasmic Reticulum Chaperone GRP78/BiP Modulates Prion Propagation *In Vitro* and *In Vivo* . Sci. Rep. 7, 44723. 10.1038/srep44723 28333162 PMC5363067

[B175] Park Y. Lee K. S. Park S. Y. Kim J. H. Kang E. Y. Kim S. W. (2015). Potential Prognostic Value of Histone Deacetylase 6 and Acetylated Heat-Shock Protein 90 in Early-Stage Breast Cancer. J. Breast Cancer 18, 249–255. 10.4048/jbc.2015.18.3.249 26472975 PMC4600689

[B176] Pavitt G. D. Ron D. (2012). New Insights into Translational Regulation in the Endoplasmic Reticulum Unfolded Protein Response. Cold Spring Harb Perspect. Biol. 4. 10.1101/cshperspect.a012278 PMC3367556 22535228

[B177] Pelham H. R. B. (1986). Speculations on the Functions of the Major Heat Shock and Glucose-Regulated Proteins. Cell 46, 959–961. 10.1016/0092-8674(86)90693-8 2944601

[B178] Piao M. J. Han X. Kang K. A. Fernando P. Herath H. Hyun J. W. (2021). The Endoplasmic Reticulum Stress Response Mediates Shikonin-Induced Apoptosis of 5-Fluorouracil-Resistant Colorectal Cancer Cells. Biomol. Ther. (Seoul) 5. 10.4062/biomolther.2021.118 PMC9047496 34607978

[B179] Pierre N. Salée C. Massot C. Blétard N. Mazzucchelli G. Smargiasso N. (2020). Proteomics Highlights Common and Distinct Pathophysiological Processes Associated with Ileal and Colonic Ulcers in Crohn's Disease. J. Crohns Colitis 14, 205–215. 10.1093/ecco-jcc/jjz130 31282946

[B180] Piszcz J. Bolkun Ł. Cichocka E. Galar M. Hołownia A. Kłoczko J. (2014). Prognostic Relevance of HSP70 Antigen and Antibody Measurement in Patients with Acute Myeloid Leukemia of Intermediate and Unfavorable Cytogenetic Risk. Polskie Archiwum Medycyny Wewnetrznej 124, 165–172. 10.20452/pamw.2184 24657921

[B270] Porter K. R. Thompson H. P. (1948). A Particulate Body Associated With Epithelial Cells Cultured From Mammary Carcinomas of Mice of a Milkfactor Strain. J. Exp. Med. 88, 15–24. 18871874 10.1084/jem.88.1.15 PMC2135805

[B272] Porter K. R. Kallman F. L. (1952). Significance of Cell Particulates as Seen by Electron Microscopy. Ann. N. Y. Acad. Sci 54, 882–891. 12976990 10.1111/j.1749-6632.1952.tb39963.x

[B181] Rees W. D. Stahl M. Jacobson K. Bressler B. Sly L. M. Vallance B. A. (2020). Enteroids Derived from Inflammatory Bowel Disease Patients Display Dysregulated Endoplasmic Reticulum Stress Pathways, Leading to Differential Inflammatory Responses and Dendritic Cell Maturation. J. Crohns Colitis 14, 948–961. 10.1093/ecco-jcc/jjz194 31796949

[B271] Qi Z. Chen L. (2019). Endoplasmic Reticulum Stress and Autophagy. Adv. Exp. Med. Biol. 1206, 167–177. 31776985 10.1007/978-981-15-0602-4_8

[B182] Renkawek K. Voorter C. E. M. Bosman G. J. C. G. M. van Workum F. P. A. de Jong W. W. (1994). Expression of αB-crystallin in Alzheimer's Disease. Acta Neuropathol. 87, 155–160. 10.1007/bf00296185 8171966

[B183] Rogalla T. Ehrnsperger M. Preville X. Kotlyarov A. Lutsch G. Ducasse C. (1999). Regulation of Hsp27 Oligomerization, Chaperone Function, and Protective Activity against Oxidative Stress/Tumor Necrosis Factor α by Phosphorylation. J. Biol. Chem. 274, 18947–18956. 10.1074/jbc.274.27.18947 10383393

[B184] Romero-Ramirez L. Cao H. Nelson D. Hammond E. Lee A.-H. Yoshida H. (2004). XBP1 Is Essential for Survival under Hypoxic Conditions and Is Required for Tumor Growth. Cancer Res. 64, 5943–5947. 10.1158/0008-5472.can-04-1606 15342372

[B185] Ron D. Habener J. F. (1992). CHOP, a Novel Developmentally Regulated Nuclear Protein that Dimerizes with Transcription Factors C/EBP and LAP and Functions as a Dominant-Negative Inhibitor of Gene Transcription. Genes Dev. 6, 439–453. 10.1101/gad.6.3.439 1547942

[B186] Ron D. (2002). Translational Control in the Endoplasmic Reticulum Stress Response. J. Clin. Invest. 110, 1383–1388. 10.1172/jci0216784 12438433 PMC151821

[B187] Ron D. Walter P. (2007). Signal Integration in the Endoplasmic Reticulum Unfolded Protein Response. Nat. Rev. Mol. Cel Biol 8, 519–529. 10.1038/nrm2199 17565364

[B188] Rouschop K. M. A. van den Beucken T. Dubois L. Niessen H. Bussink J. Savelkouls K. (2010). The Unfolded Protein Response Protects Human Tumor Cells during Hypoxia through Regulation of the Autophagy Genes MAP1LC3B and ATG5. J. Clin. Invest. 120, 127–141. 10.1172/jci40027 20038797 PMC2798689

[B189] Rutkowski D. T. Hegde R. S. (2010). Regulation of Basal Cellular Physiology by the Homeostatic Unfolded Protein Response. J. Cel. Biol. 189, 783–794. 10.1083/jcb.201003138 PMC2878945 20513765

[B190] Ryan D. Carberry S. Murphy Á. C. Lindner A. U. Fay J. Hector S. (2016). Calnexin, an ER-Induced Protein, Is a Prognostic Marker and Potential Therapeutic Target in Colorectal Cancer. J. Transl Med. 14, 196. 10.1186/s12967-016-0948-z 27369741 PMC4930591

[B191] Sakitani K. Hirata Y. Hikiba Y. Hayakawa Y. Ihara S. Suzuki H. (2015). Inhibition of Autophagy Exerts Anti-colon Cancer Effects via Apoptosis Induced by P53 Activation and ER Stress. BMC Cancer 15, 795. 10.1186/s12885-015-1789-5 26496833 PMC4620020

[B192] Salaroglio I. C. Panada E. Moiso E. Buondonno I. Provero P. Rubinstein M. (2017). PERK Induces Resistance to Cell Death Elicited by Endoplasmic Reticulum Stress and Chemotherapy. Mol. Cancer 16, 91. 10.1186/s12943-017-0657-0 28499449 PMC5427528

[B193] Salas M. Tuchweber B. Kourounakis P. (1980). Liver Ultrastructure during Acute Stress. Pathol. - Res. Pract. 167, 217–233. 10.1016/s0344-0338(80)80052-5 7433233

[B194] Samali A. Robertson J. D. Peterson E. Manero F. van Zeijl L. Paul C. (2001). Hsp27 Protects Mitochondria of Thermotolerant Cells against Apoptotic Stimuli. Cell Stress Chaper 6, 49–58. 10.1379/1466-1268(2001)006<0049:hpmotc>2.0.co;2 PMC434383 11525243

[B195] Samoila I. Dinescu S. Costache M. (2020). Interplay between Cellular and Molecular Mechanisms Underlying Inflammatory Bowel Diseases Development-A Focus on Ulcerative Colitis. Cells 9. 10.3390/cells9071647 PMC7408467 32659925

[B196] Sanches M. Duffy N. M. Talukdar M. Thevakumaran N. Chiovitti D. Canny M. D. (2014). Structure and Mechanism of Action of the Hydroxy-Aryl-Aldehyde Class of IRE1 Endoribonuclease Inhibitors. Nat. Commun. 5, 4202. 10.1038/ncomms5202 25164867 PMC4486471

[B197] Santel A. Fuller M. T. (2001). Control of Mitochondrial Morphology by a Human Mitofusin. J. Cel Sci 114, 867–874. 10.1242/jcs.114.5.867 11181170

[B198] Scheuner D. Song B. McEwen E. Liu C. Laybutt R. Gillespie P. (2001). Translational Control Is Required for the Unfolded Protein Response and *In Vivo* Glucose Homeostasis. Mol. Cel. 7, 1165–1176. 10.1016/s1097-2765(01)00265-9 11430820

[B199] Schroder K. Tschopp J. (2010). The Inflammasomes. Cell 140, 821–832. 10.1016/j.cell.2010.01.040 20303873

[B200] Scorrano L. Oakes S. A. Opferman J. T. Cheng E. H. Sorcinelli M. D. Pozzan T. (2003). BAX and BAK Regulation of Endoplasmic Reticulum Ca 2+ : A Control Point for Apoptosis. Science 300, 135–139. 10.1126/science.1081208 12624178

[B201] Shen J. Chen X. Hendershot L. Prywes R. (2002). ER Stress Regulation of ATF6 Localization by Dissociation of BiP/GRP78 Binding and Unmasking of Golgi Localization Signals. Developmental Cel. 3, 99–111. 10.1016/s1534-5807(02)00203-4 12110171

[B202] Shi Y. Vattem K. M. Sood R. An J. Liang J. Stramm L. (1998). Identification and Characterization of Pancreatic Eukaryotic Initiation Factor 2 α-Subunit Kinase, PEK, Involved in Translational Control. Mol. Cel Biol 18, 7499–7509. 10.1128/mcb.18.12.7499 PMC109330 9819435

[B203] Shi Z. Yu X. Yuan M. Lv W. Feng T. Bai R. (2019). Activation of the PERK-ATF4 Pathway Promotes Chemo-Resistance in colon Cancer Cells. Sci. Rep. 9, 3210. 10.1038/s41598-019-39547-x 30824833 PMC6397152

[B204] Shibue T. Taniguchi T. (2006). BH3-only Proteins: Integrated Control point of Apoptosis. Int. J. Cancer 119, 2036–2043. 10.1002/ijc.21751 16572413

[B205] Sidrauski C. Walter P. (1997). The Transmembrane Kinase Ire1p Is a Site-specific Endonuclease that Initiates mRNA Splicing in the Unfolded Protein Response. Cell 90, 1031–1039. 10.1016/s0092-8674(00)80369-4 9323131

[B206] Sitia R. Braakman I. (2003). Quality Control in the Endoplasmic Reticulum Protein Factory. Nature 426, 891–894. 10.1038/nature02262 14685249

[B207] Sokka A.-L. Putkonen N. Mudo G. Pryazhnikov E. Reijonen S. Khiroug L. (2007). Endoplasmic Reticulum Stress Inhibition Protects against Excitotoxic Neuronal Injury in the Rat Brain. J. Neurosci. 27, 901–908. 10.1523/jneurosci.4289-06.2007 17251432 PMC6672923

[B209] Sprague-Piercy M. A. Rocha M. A. Kwok A. O. Martin R. W. (2021). α-Crystallins in the Vertebrate Eye Lens: Complex Oligomers and Molecular Chaperones. Annu. Rev. Phys. Chem. 72, 143–163. 10.1146/annurev-physchem-090419-121428 33321054 PMC8062273

[B210] Stengel S. T. Fazio A. Lipinski S. Jahn M. T. Aden K. Ito G. (2020). Activating Transcription Factor 6 Mediates Inflammatory Signals in Intestinal Epithelial Cells upon Endoplasmic Reticulum Stress. Gastroenterology 159, 1357–1374. e1310. 10.1053/j.gastro.2020.06.088 32673694 PMC7923714

[B211] Su Q. Wang S. Gao H. Q. Kazemi S. Harding H. P. Ron D. (2008). Modulation of the Eukaryotic Initiation Factor 2 α-Subunit Kinase PERK by Tyrosine Phosphorylation. J. Biol. Chem. 283, 469–475. 10.1074/jbc.m704612200 17998206

[B212] Su Y. Li F. (2016). Endoplasmic Reticulum Stress in Brain Ischemia. Int. J. Neurosci. 126, 681–691. 10.3109/00207454.2015.1059836 26289799

[B213] Suetsugu S. Toyooka K. Senju Y. (2010). Subcellular Membrane Curvature Mediated by the BAR Domain Superfamily Proteins. Semin. Cel Developmental Biol. 21, 340–349. 10.1016/j.semcdb.2009.12.002 19963073

[B214] Sun H. Lin D.-C. Guo X. Masouleh B. K. Gery S. Cao Q. (2016). Inhibition of IRE1α-Driven Pro-survival Pathways Is a Promising Therapeutic Application in Acute Myeloid Leukemia. Oncotarget 7, 18736–18749. 10.18632/oncotarget.7702 26934650 PMC4951325

[B215] Šuštić T. van Wageningen S. Bosdriesz E. Reid R. J. D. Dittmar J. Lieftink C. (2018). A Role for the Unfolded Protein Response Stress Sensor ERN1 in Regulating the Response to MEK Inhibitors in KRAS Mutant colon Cancers. Genome Med. 10, 90. 10.1186/s13073-018-0600-z 30482246 PMC6258447

[B216] Szegezdi E. Logue S. E. Gorman A. M. Samali A. (2006). Mediators of Endoplasmic Reticulum Stress‐induced Apoptosis. EMBO Rep. 7, 880–885. 10.1038/sj.embor.7400779 16953201 PMC1559676

[B217] Tabas I. Ron D. (2011). Integrating the Mechanisms of Apoptosis Induced by Endoplasmic Reticulum Stress. Nat. Cel Biol 13, 184–190. 10.1038/ncb0311-184 PMC3107571 21364565

[B218] Talloczy Z. Jiang W. Virgin H. W. Leib D. A. Scheuner D. Kaufman R. J. (2002). Regulation of Starvation- and Virus-Induced Autophagy by the eIF2 Kinase Signaling Pathway. Proc. Natl. Acad. Sci. 99, 190–195. 10.1073/pnas.012485299 11756670 PMC117537

[B219] Thuringer D. Berthenet K. Cronier L. Solary E. Garrido C. (2015). Primary Tumor- and Metastasis-Derived colon Cancer Cells Differently Modulate Connexin Expression and Function in Human Capillary Endothelial Cells. Oncotarget 6, 28800–28815. 10.18632/oncotarget.4894 26320187 PMC4745693

[B220] Tirasophon W. Welihinda A. A. Kaufman R. J. (1998). A Stress Response Pathway from the Endoplasmic Reticulum to the Nucleus Requires a Novel Bifunctional Protein Kinase/endoribonuclease (Ire1p) in Mammalian Cells. Genes Dev. 12, 1812–1824. 10.1101/gad.12.12.1812 9637683 PMC316900

[B221] Todd D. J. Lee A.-H. Glimcher L. H. (2008). The Endoplasmic Reticulum Stress Response in Immunity and Autoimmunity. Nat. Rev. Immunol. 8, 663–674. 10.1038/nri2359 18670423

[B222] Trabucchi E. Mukenge S. Baratti C. Colombo R. Fregoni F. Montorsi W. (1986). Differential Diagnosis of Crohn's Disease of the colon from Ulcerative Colitis: Ultrastructure Study with the Scanning Electron Microscope. Int. J. Tissue React. 8, 79–84. 3949447

[B223] Tsuru A. Fujimoto N. Takahashi S. Saito M. Nakamura D. Iwano M. (2013). Negative Feedback by IRE1 Optimizes Mucin Production in Goblet Cells. Proc. Natl. Acad. Sci. 110, 2864–2869. 10.1073/pnas.1212484110 23386727 PMC3581977

[B224] Tung Y.-C. Tsai M.-L. Kuo F.-L. Lai C.-S. Badmaev V. Ho C.-T. (2015). Se-Methyl-L-selenocysteine Induces Apoptosis via Endoplasmic Reticulum Stress and the Death Receptor Pathway in Human Colon Adenocarcinoma COLO 205 Cells. J. Agric. Food Chem. 63, 5008–5016. 10.1021/acs.jafc.5b01779 25943382

[B225] Tytgat K. M. A. J. van der Wal J.-W. G. Einerhand A. W. C. Büller H. A. Dekker J. (1996). Quantitative Analysis of MUC2 Synthesis in Ulcerative Colitis. Biochem. Biophysical Res. Commun. 224, 397–405. 10.1006/bbrc.1996.1039 8702401

[B226] Urano F. Wang X. Bertolotti A. Zhang Y. Chung P. Harding H. P. (2000). Coupling of Stress in the ER to Activation of JNK Protein Kinases by Transmembrane Protein Kinase IRE1. Science 287, 664–666. 10.1126/science.287.5453.664 10650002

[B227] van de Veerdonk F. L. Netea M. G. Dinarello C. A. Joosten L. A. B. (2011). Inflammasome Activation and IL-1β and IL-18 Processing during Infection. Trends Immunology 32, 110–116. 10.1016/j.it.2011.01.003 21333600

[B228] Van Klinken B. J.-W. Van der Wal J.-W. G. Einerhand A. W. C. Büller H. A. Dekker J. (1999). Sulphation and Secretion of the Predominant Secretory Human Colonic Mucin MUC2 in Ulcerative Colitis. Gut 44, 387–393. 10.1136/gut.44.3.387 10026326 PMC1727426

[B229] Vance J. E. (2015). Phospholipid Synthesis and Transport in Mammalian Cells. Traffic 16, 1–18. 10.1111/tra.12230 25243850

[B230] Vannuvel K. Renard P. Raes M. Arnould T. (2013). Functional and Morphological Impact of ER Stress on Mitochondria. J. Cel. Physiol. 228, 1802–1818. 10.1002/jcp.24360 23629871

[B231] Vattem K. M. Wek R. C. (2004). Reinitiation Involving Upstream ORFs Regulates ATF4 mRNA Translation in Mammalian Cells. Proc. Natl. Acad. Sci. 101, 11269–11274. 10.1073/pnas.0400541101 15277680 PMC509193

[B232] Verfaillie T. Rubio N. Garg A. D. Bultynck G. Rizzuto R. Decuypere J.-P. (2012). PERK Is Required at the ER-Mitochondrial Contact Sites to Convey Apoptosis after ROS-Based ER Stress. Cel Death Differ 19, 1880–1891. 10.1038/cdd.2012.74 PMC3469056 22705852

[B233] Vieujean S. Hu S. Bequet E. Salee C. Massot C. Bletard N. (2021). Potential Role of Epithelial Endoplasmic Reticulum Stress and Anterior Gradient Protein 2 Homologue in Crohn's Disease Fibrosis. J. Crohns Colitis 15, 1737–1750. 10.1093/ecco-jcc/jjab061 33822017 PMC8861373

[B234] Volkmann K. Lucas J. L. Vuga D. Wang X. Brumm D. Stiles C. (2011). Potent and Selective Inhibitors of the Inositol-Requiring Enzyme 1 Endoribonuclease. J. Biol. Chem. 286, 12743–12755. 10.1074/jbc.m110.199737 21303903 PMC3069474

[B235] Vos M. J. Hageman J. Carra S. Kampinga H. H. (2008). Structural and Functional Diversities between Members of the Human HSPB, HSPH, HSPA, and DNAJ Chaperone Families. Biochemistry 47, 7001–7011. 10.1021/bi800639z 18557634

[B236] Walter P. Ron D. (2011). The Unfolded Protein Response: from Stress Pathway to Homeostatic Regulation. Science 334, 1081–1086. 10.1126/science.1209038 22116877

[B237] Wang A. Liu X. Sheng S. Ye H. Peng T. Shi F. (2009). Dysregulation of Heat Shock Protein 27 Expression in Oral Tongue Squamous Cell Carcinoma. BMC cancer 9, 167. 10.1186/1471-2407-9-167 19497117 PMC2696470

[B238] Wang C. Y. Guo S. T. Croft A. Yan X. G. Jin L. Zhang X. D. (2018). BAG3-dependent Expression of Mcl-1 Confers Resistance of mutantKRAScolon Cancer Cells to the HSP90 Inhibitor AUY922. Mol. Carcinog 57, 284–294. 10.1002/mc.22755 29068469

[B239] Wang Q. Liu M. Chen Y. Xu L. Wu B. Wu Y. (2019a). Muscovy Duck Reovirus p10.8 Protein Induces ER Stress and Apoptosis through the Bip/IRE1/XBP1 Pathway. Vet. Microbiol. 228, 234–245. 10.1016/j.vetmic.2018.12.011 30593373

[B240] Wang S. Zhang X. Wang H. Wang Y. Chen P. Wang L. (2020). Heat Shock Protein 27 Enhances SUMOylation of Heat Shock Protein B8 to Accelerate the Progression of Breast Cancer. Am. J. Pathol. 190, 2464–2477. 10.1016/j.ajpath.2020.04.012 33222991

[B241] Wang X.-Z. Harding H. P. Zhang Y. Jolicoeur E. M. Kuroda M. Ron D. (1998). Cloning of Mammalian Ire1 Reveals Diversity in the ER Stress Responses. EMBO J. 17, 5708–5717. 10.1093/emboj/17.19.5708 9755171 PMC1170899

[B242] Wang X. Z. Lawson B. Brewer J. W. Zinszner H. Sanjay A. Mi L. J. (1996). Signals from the Stressed Endoplasmic Reticulum Induce C/EBP-homologous Protein (CHOP/GADD153). Mol. Cel Biol 16, 4273–4280. 10.1128/mcb.16.8.4273 PMC231426 8754828

[B243] Wang Y. Alam G. N. Ning Y. Visioli F. Dong Z. Nör J. E. (2012). The Unfolded Protein Response Induces the Angiogenic Switch in Human Tumor Cells through the PERK/ATF4 Pathway. Cancer Res. 72, 5396–5406. 10.1158/0008-5472.can-12-0474 22915762 PMC3743425

[B244] Wang Y. Chiang I.-L. Ohara T. E. Fujii S. Cheng J. Muegge B. D. (2019b). Long-Term Culture Captures Injury-Repair Cycles of Colonic Stem Cells. Cell 179, 1144–1159. 10.1016/j.cell.2019.10.015 31708126 PMC6904908

[B245] Wang Y. Shen J. Arenzana N. Tirasophon W. Kaufman R. J. Prywes R. (2000). Activation of ATF6 and an ATF6 DNA Binding Site by the Endoplasmic Reticulum Stress Response. J. Biol. Chem. 275, 27013–27020. 10.1016/s0021-9258(19)61473-0 10856300

[B246] Wei M. C. Zong W.-X. Cheng E. H.-Y. Lindsten T. Panoutsakopoulou V. Ross A. J. (2001). Proapoptotic BAX and BAK: a Requisite Gateway to Mitochondrial Dysfunction and Death. Science 292, 727–730. 10.1126/science.1059108 11326099 PMC3049805

[B247] Welch W. J. Garrels J. I. Thomas G. P. Lin J. J. Feramisco J. R. (1983). Biochemical Characterization of the Mammalian Stress Proteins and Identification of Two Stress Proteins as Glucose- and Ca2+-Ionophore-Regulated Proteins. J. Biol. Chem. 258, 7102–7111. 10.1016/s0021-9258(18)32338-x 6406494

[B248] Wieckowski M. R. Giorgi C. Lebiedzinska M. Duszynski J. Pinton P. (2009). Isolation of Mitochondria-Associated Membranes and Mitochondria from Animal Tissues and Cells. Nat. Protoc. 4, 1582–1590. 10.1038/nprot.2009.151 19816421

[B249] Wielenga M. C. B. Colak S. Heijmans J. van Lidth de Jeude J. F. Rodermond H. M. Paton J. C. (2015). ER-Stress-Induced Differentiation Sensitizes Colon Cancer Stem Cells to Chemotherapy. Cel Rep. 13, 489–494. 10.1016/j.celrep.2015.09.016 26456824

[B250] Wu C. Xu H. Li J. Hu X. Wang X. Huang Y. (2020). Baicalein Attenuates Pyroptosis and Endoplasmic Reticulum Stress Following Spinal Cord Ischemia-Reperfusion Injury via Autophagy Enhancement. Front. Pharmacol. 11, 1076. 10.3389/fphar.2020.01076 32903577 PMC7438740

[B251] Wu P. Zhang H. Qi L. Tang Q. Tang Y. Xie Z. (2012). Identification of ERp29 as a Biomarker for Predicting Nasopharyngeal Carcinoma Response to Radiotherapy. Oncol. Rep. 27, 987–994. 10.3892/or.2011.1586 22160175 PMC3583588

[B252] Xia X. Wang X. Zheng Y. Jiang J. Hu J. (2019). What Role Does Pyroptosis Play in Microbial Infection? J. Cel Physiol 234, 7885–7892. 10.1002/jcp.27909 30537070

[B253] Xu X. Li Q. Li L. Zeng M. Zhou X. Cheng Z. (2021). Endoplasmic Reticulum stress/XBP1 Promotes Airway Mucin Secretion under the Influence of Neutrophil Elastase. Int. J. Mol. Med. 47. 10.3892/ijmm.2021.4914 PMC7979262 33760106

[B254] Xu Y. Chen Z. Zhang G. Xi Y. Sun R. Wang X. (2016). HSP90B1 Overexpression Predicts Poor Prognosis in NSCLC Patients. Tumor Biol. 37, 14321–14328. 10.1007/s13277-016-5304-7 27599983

[B255] Yang X. Srivastava R. Howell S. H. Bassham D. C. (2016). Activation of Autophagy by Unfolded Proteins during Endoplasmic Reticulum Stress. Plant J. 85, 83–95. 10.1111/tpj.13091 26616142

[B256] Ye C. Dickman M. B. Whitham S. A. Payton M. Verchot J. (2011). The Unfolded Protein Response Is Triggered by a Plant Viral Movement Protein. Plant Physiol. 156, 741–755. 10.1104/pp.111.174110 21474436 PMC3177272

[B257] Yin S. Li L. Tao Y. Yu J. Wei S. Liu M. (2021). The Inhibitory Effect of Artesunate on Excessive Endoplasmic Reticulum Stress Alleviates Experimental Colitis in Mice. Front. Pharmacol. 12, 629798. 10.3389/fphar.2021.629798 33767628 PMC7985062

[B258] Yoshida H. Haze K. Yanagi H. Yura T. Mori K. (1998). Identification of the Cis-Acting Endoplasmic Reticulum Stress Response Element Responsible for Transcriptional Induction of Mammalian Glucose-Regulated Proteins. J. Biol. Chem. 273, 33741–33749. 10.1074/jbc.273.50.33741 9837962

[B259] Yoshida H. Matsui T. Yamamoto A. Okada T. Mori K. (2001). XBP1 mRNA Is Induced by ATF6 and Spliced by IRE1 in Response to ER Stress to Produce a Highly Active Transcription Factor. Cell 107, 881–891. 10.1016/s0092-8674(01)00611-0 11779464

[B260] Yu Z. Zhi J. Peng X. Zhong X. Xu A. (2010). Clinical Significance of HSP27 Expression in Colorectal Cancer. Mol. Med. Rep. 3, 953–958. 10.3892/mmr.2010.372 21472339

[B261] Zhang B. Su X. Xie Z. Ding H. Wang T. Xie R. (2021a). Inositol-Requiring Kinase 1 Regulates Apoptosis via Inducing Endoplasmic Reticulum Stress in Colitis Epithelial Cells. Dig. Dis. Sci. 66, 3015–3025. 10.1007/s10620-020-06622-7 33043405

[B262] Zhang H. S. Chen Y. Fan L. Xi Q. L. Wu G. H. Li X. X. (2015). The Endoplasmic Reticulum Stress Sensor IRE1α in Intestinal Epithelial Cells Is Essential for Protecting against Colitis. J. Biol. Chem. 290, 15327–15336. 10.1074/jbc.m114.633560 25925952 PMC4463471

[B263] Zhang L. Nosak C. Sollazzo P. Odisho T. Volchuk A. (2014). IRE1 Inhibition Perturbs the Unfolded Protein Response in a Pancreatic β-cell Line Expressing Mutant Proinsulin, but Does Not Sensitize the Cells to Apoptosis. BMC Cel Biol 15, 29. 10.1186/1471-2121-15-29 PMC4118655 25011481

[B264] Zhang Y. Liu Y. Zhou Y. Zheng Z. Tang W. Song M. (2021b). Lentinan Inhibited colon Cancer Growth by Inducing Endoplasmic Reticulum Stress-Mediated Autophagic Cell Death and Apoptosis. Carbohydr. Polym. 267, 118154. 10.1016/j.carbpol.2021.118154 34119128

[B265] Zhao F. Edwards R. Dizon D. Afrasiabi K. Mastroianni J. R. Geyfman M. (2010). Disruption of Paneth and Goblet Cell Homeostasis and Increased Endoplasmic Reticulum Stress in Agr2^−/−^ Mice. Developmental Biol. 338, 270–279. 10.1016/j.ydbio.2009.12.008 PMC2937056 20025862

[B266] Zhou Y. Tong Z. Jiang S. Zheng W. Zhao J. Zhou X. (2020). The Roles of Endoplasmic Reticulum in NLRP3 Inflammasome Activation. Cells 9. 10.3390/cells9051219 PMC7291288 32423023

[B267] Zinszner H. Kuroda M. Wang X. Batchvarova N. Lightfoot R. T. Remotti H. (1998). CHOP Is Implicated in Programmed Cell Death in Response to Impaired Function of the Endoplasmic Reticulum. Genes Development 12, 982–995. 10.1101/gad.12.7.982 9531536 PMC316680

[B268] Zong W.-X. Li C. Hatzivassiliou G. Lindsten T. Yu Q.-C. Yuan J. (2003). Bax and Bak Can Localize to the Endoplasmic Reticulum to Initiate Apoptosis. J. Cel. Biol. 162, 59–69. 10.1083/jcb.200302084 PMC2172724 12847083

[B269] Zou H. Limpert A. S. Zou J. Dembo A. Lee P.-S. Grant D. (2015). Benzodiazepinone Derivatives Protect against Endoplasmic Reticulum Stress-Mediated Cell Death in Human Neuronal Cell Lines. ACS Chem. Neurosci. 6, 464–475. 10.1021/cn500297v 25544056 PMC4368043

