# Corrigendum: Endoplasmic reticulum stress of gut enterocyte and intestinal diseases

**DOI:** 10.3389/fmolb.2022.1051738

**Published:** 2022-10-25

**Authors:** Han Gao, Chengwei He, Rongxuan Hua, Yuexin Guo, Boya Wang, Chen Liang, Lei Gao, Hongwei Shang, Jing-Dong Xu

**Affiliations:** ^1^ Department of Physiology and Pathophysiology, School of Basic Medical Sciences, Capital Medical University, Beijing, China; ^2^ Department of Clinical Medicine, School of Basic Medical Sciences, Capital Medical University, Beijing, China; ^3^ Department of Oral Medicine, School of Basic Medical Sciences, Capital Medical University, Beijing, China; ^4^ Undergraduate Student of 2018 Eight Program of Clinical Medicine, Peking University Health Science Center, Beijing, China; ^5^ Department of Biomedical Informatics, School of Biomedical Engineering, Capital Medical University, Beijing, China; ^6^ Experimental Center for Morphological Research Platform, Capital Medical University, Beijing, China

**Keywords:** endoplasmic reticulum stress, unfolded protein response, cell death, colon cancer, treatment target, inflammatory bowel disease

In the published article, there were errors in the following affiliations:

Instead of “Department of Physiology and Pathophysiology, Basic Medical College, Capital Medical University, Beijing, China”, it should be “Department of Physiology and Pathophysiology, School of Basic Medical Sciences, Capital Medical University, Beijing, China”.

Instead of “Department of Clinical Medicine, Basic Medical College, Capital Medical University, Beijing, China”, it should be “Department of Clinical Medicine, School of Basic Medical Sciences, Capital Medical University, Beijing, China”.

Instead of “Department of Oral Medicine, Basic Medical College, Capital Medical University, Beijing, China”, it should be “Department of Oral Medicine, School of Basic Medical Sciences, Capital Medical University, Beijing, China”.

The authors apologize for this error and state that this does not change the scientific conclusions of the article in any way. The original article has been updated.

